# A New Model to Produce Infectious Hepatitis C Virus without the Replication Requirement

**DOI:** 10.1371/journal.ppat.1001333

**Published:** 2011-04-14

**Authors:** Miriam Triyatni, Edward A. Berger, Bertrand Saunier

**Affiliations:** 1 Molecular Structure Section, Laboratory of Viral Diseases, NIAID, NIH, Bethesda, Maryland, United States of America; 2 Paris-Descartes University, Faculty of Medicine, Paris, France; 3 Institut Cochin, Paris, France; 4 Inserm U1016, Paris, France; University of Southern California, United States of America

## Abstract

Numerous constraints significantly hamper the experimental study of hepatitis C virus (HCV). Robust replication in cell culture occurs with only a few strains, and is invariably accompanied by adaptive mutations that impair *in vivo* infectivity/replication. This problem complicates the production and study of authentic HCV, including the most prevalent and clinically important genotype 1 (subtypes 1a and 1b). Here we describe a novel cell culture approach to generate infectious HCV virions without the HCV replication requirement and the associated cell-adaptive mutations. The system is based on our finding that the intracellular environment generated by a West-Nile virus (WNV) subgenomic replicon rendered a mammalian cell line permissive for assembly and release of infectious HCV particles, wherein the HCV RNA with correct 5′ and 3′ termini was produced in the cytoplasm by a plasmid-driven dual bacteriophage RNA polymerase-based transcription/amplification system. The released particles preferentially contained the HCV-based RNA compared to the WNV subgenomic RNA. Several variations of this system are described with different HCV-based RNAs: (i) HCV bicistronic particles (HCVbp) containing RNA encoding the HCV structural genes upstream of a cell-adapted subgenomic replicon, (ii) HCV reporter particles (HCVrp) containing RNA encoding the bacteriophage SP6 RNA polymerase in place of HCV nonstructural genes, and (iii) HCV wild-type particles (HCVwt) containing unmodified RNA genomes of diverse genotypes (1a, strain H77; 1b, strain Con1; 2a, strain JFH-1). Infectivity was assessed based on the signals generated by the HCV RNA molecules introduced into the cytoplasm of target cells upon virus entry, i.e. HCV RNA replication and protein production for HCVbp in Huh-7.5 cells as well as for HCVwt in HepG2-CD81 cells and human liver slices, and SP6 RNA polymerase-driven firefly luciferase for HCVrp in target cells displaying candidate HCV surface receptors. HCV infectivity was inhibited by pre-incubation of the particles with anti-HCV antibodies and by a treatment of the target cells with leukocyte interferon plus ribavirin. The production of authentic infectious HCV particles of virtually any genotype without the adaptive mutations associated with *in vitro* HCV replication represents a new paradigm to decipher the requirements for HCV assembly, release, and entry, amenable to analyses of wild type and genetically modified viruses of the most clinically significant HCV genotypes.

## Author Summary

Two decades after its identification, hepatitis C virus (HCV) remains a leading cause of serious liver diseases worldwide. The poor *in vitro* propagation of patient isolates has impaired their study. Conversely, viral strains of the most prevalent (∼70% of total infections) and clinically problematic (∼45% cured with the standard of care) genotype 1 adapted for *in vitro* replication display mutations impairing yield and/or *in vivo* infectivity. We established a new cell culture model for producing infectious HCV in a cell line stably bearing a subgenomic replicon from West Nile virus (a flavivirus belonging to the same family as HCV) that circumvents the requirement for HCV RNA replication. To study viral infectivity *in vitro*, we devised several HCV genome-based constructs. This system produced wild type HCV particles of subtypes 1a, 1b, 2a and a 1b/2a chimera. All specifically infected permissive target cells, and HCV particles containing wild type genomes known to be infectious *in vivo* infected human liver slices *ex vivo*. The production of authentic HCV particles independent of HCV RNA replication represents a new paradigm to decipher requirements for HCV assembly, release, and entry, amenable to analyses of wild type and genetically modified viruses of the most clinically significant genotypes.

## Introduction

HCV infects 2–3% of the world population. A majority of infected people fail to clear the virus and are at risk for developing serious liver complications (reviewed in [1]). HCV belongs to the genus *Hepacivirus* in the *Flaviviridæ* family, and at least six genotypes have been identified so far [2]. Greater than two thirds of HCV infections diagnosed worldwide are of subtypes 1a or 1b [2]. There is no approved vaccine and available treatments are much less effective against genotype 1 compared to other genotypes. The limited experimental availability of chimpanzees, the primary animal model for HCV [3], [4], and difficulties encountered in reproducing true infection in small animals have significantly limited the use of *in vivo* models to study the biology of this virus. The structure of the intact virion is unknown, and it is still unclear how the RNA genome [5] circulates in infected patients. In addition, although the natural target cells of HCV are primarily hepatocytes in the liver, *in vitro* most human hepatic cells poorly propagate HCV isolates from patients (e.g. [6]). *In vitro* studies were nevertheless marked by two breakthroughs allowing for the screening of new antiviral compounds. First, subgenomic replicons (i.e. without structural genes) of subtypes 1b [7], [8] and 1a [9] were established in selected subclones of the human hepatic Huh-7 cell line that are highly permissive for HCV replication, e.g. Huh-7.5 cells [10]. Subsequently, a full infectious cycle was reproduced in cell culture with JFH-1, a particular strain of genotype 2a [11], [12], or with a J6/JFH-1 chimera [13]; the released particles are referred to as HCVcc.

Although propagation of a few HCV strains in replication-permissive cell lines has been an important contribution to the field, it has long been recognized that these models are complicated by the particularly high error rate of the HCV RNA replicase [14]. Combined with the *in vitro* selective pressure, e.g. associated with the modifications acquired by the permissive cell lines [15], or viral recombination between genotypes [16]–[18], it inevitably results in the emergence of adaptive/escape variants [19]. However, cell culture-adapted HCV most often displays lack of infectivity or impaired fitness *in vivo* [20], [21]. Conversely, HCV genomes with a consensus sequence that are infectious in chimpanzees are not infectious in cell culture, e.g. in Huh-7.5 cells [9], [22]. This issue is especially perplexing with genotype 1 strains, for which the accumulation of cell-adaptive mutations that enhance its RNA replication results at best in low yields of HCVcc with impaired infectivity [19], [23]. Intergenotypic JFH-1 chimeras have been engineered to tentatively overcome such limitations [16]–[18] but have been shown to accumulate structural gene compensatory mutations [16]. As such mutations and their associated complications result from the viral RNA replication process, we reasoned that uncoupling the production of infectious HCV particles from HCV RNA replication would circumvent major limitations associated with existing *in vitro* systems requiring such coupling.

All known *Flaviviridæ* members replicate in the cytoplasm of their target cells and induce membrane rearrangements mostly deriving from the endoplasmic reticulum (ER) [24], [25]. Strongly connected to RNA replication [26], assembly of infectious flavivirus particles occurs within a distinct sub-compartment of rearranged membranes [27], [28]. It has been possible to produce flavivirus virions by providing their structural genes in *trans*. Thus, upon expression of WNV structural genes: core, pre-membrane (prM) and envelope (E), baby hamster kidney (BHK)-21 cells carrying a WNV subgenomic replicon encoding a reporter gene release infectious WNV reporter-particles (WNVrp) containing subgenomic replicon RNA [29], [30]. Although distantly related within the *Flaviviridæ* family, the *Flavivirus* and *Hepacivirus genera* display common features [31]. We therefore examined whether, as for WNV, infectious HCV particles could be formed when the structural proteins are encoded in *trans*. While we did not observe such *trans*-complementation in a HCV replication-permissive cell line, we made the surprising observation that non-hepatic mammalian cells previously used to study the biology of *Flaviviridæ* (including HCV) and bearing a flavivirus subgenomic replicon can produce infectious HCV of diverse genotypes from genomic RNA produced by a plasmid-based system involving cytoplasmic transcription by bacteriophage T7 RNA polymerase. The lack of involvement of the HCV RNA replication machinery avoids the occurrence of cell-adaptive mutations in the HCV genomes.

## Results

### Assembly and release of HCV particles by BHK-WNV cells

**Figure 1:**
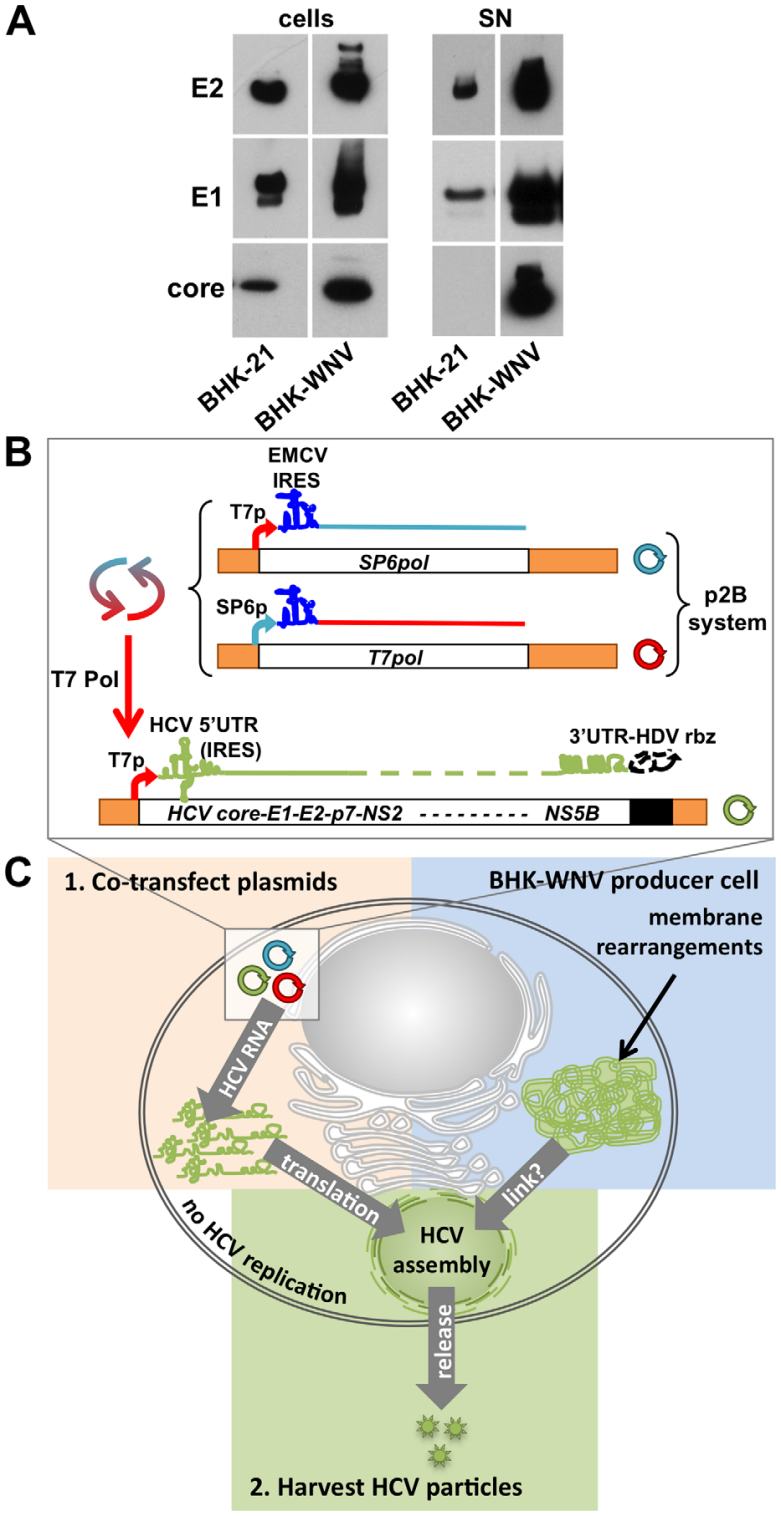
WNV subgenomic replicon enhances the release of HCV particles. (A) HCV structural genes (core to p7, driven by a CMV promoter) were expressed in BHK-21 or BHK-WNV cells. Three days later, equal amounts of lysates (cells) and SN (corresponding to pellets from 15 ml culture media ultracentrifuged through 15% sucrose cushion) from both cell types were loaded onto a gel, and HCV proteins (E2, E1 and core) were analyzed by Western blot. (B) System of plasmids to produce HCV particles in BHK-WNV cells: the plasmid-driven dual bacteriophage RNA polymerase-based transcription/amplification (p2B) system consists of two plasmids (cf. Materials and Methods) producing T7 and Sp6 bacteriophage DNA-dependent RNA polymerases (DdRp) that cross-amplify each other's transcription and were used to generate uncapped RNA with T7 promoter-driven HCV-encoding plasmids in the cytoplasm of producer cells. HDV rbz = hepatitis delta virus antisense ribozyme; T7 Pol = T7 DdRp; T7*pol* (or SP6*pol*) = T7 (or SP6) DdRp genes; T7p (or SP6p) = T7 (or SP6 Pol) cognate promoter. (C) Cartoon depicting the main components involved in the system to produce infectious HCV particle by BHK-WNV cells: *left* (orange), the intracytoplasmic production of T7 Pol-transcribed HCV genomic RNA; *right* (blue), cellular changes induced by the stably established WNV subgenomic replicon, including intracytoplasmic membrane rearrangements; *bottom* (green), HCV assembly and release.

In initial analyses of the possible effects of flavivirus replicons on HCV virus particle production from proteins provided in *trans*, we observed that release of HCV structural proteins (expressed from a cytomegalovirus immediate early promoter and harvested by ultracentrifugation) was dramatically enhanced in BHK-21 cells carrying a lineage II WNV subgenomic replicon [32] (referred to as BHK-WNV cells in this study) compared to parental cells; a less pronounced increase was observed in the cell lysate (Fig. 1A). This result suggests that, in the complete absence of HCV RNA replication, the WNV subgenomic replicon had generated a permissive environment for releasing HCV particles. Surprisingly, these effects were not observed in the seemingly more relevant Huh-7.5 human hepatocyte cell line, in which we found that the presence of an HCV subgenomic replicon inhibited rather than stimulated release of HCV structural proteins (both of genotype 1a) provided in *trans* (Fig. S1A). Moreover, we were unable to stably establish an HCV subgenomic replicon in BHK-21 cells. Based on these results, we considered the potential of the BHK-WNV cell system to produce infectious HCV particles if appropriate HCV-based RNA molecules were generated in the cytoplasm. Such a system might potentially enable virus production of the most prevalent but experimentally difficult genotype 1 strains.

### Model system for production of infectious HCV particles in BHK-WNV cells

To test this hypothesis, we devised a strategy for generating HCV-based RNA molecules in the cytoplasm of BHK-WNV cells (Fig. 1B). One component of this approach consisted of a dual-plasmid bacteriophage polymerase (p2B) system consisting of the genes for the DNA-dependent RNA polymerases from both bacteriophages T7 and SP6 (*T7pol* and *SP6pol*, respectively), each linked to their reciprocal promoter. The other component was a plasmid encoding the HCV genomic sequence of interest flanked at the 5′ end by the bacteriophage T7 promoter, and at the 3′ end by a hepatitis delta virus antisense ribozyme (*HDVrbz*; cf. Materials and Methods). We reasoned that co-transfection of these two components into BHK-WNV cells would result in cytoplasmic co-amplification of both bacteriophage polymerases; T7 Pol would then drive high level cytoplasmic production of uncapped HCV genomic RNA with correct 3′ termini (by HDV rbz self-cleavage) that would serve as template for translation of HCV proteins (driven by the HCV IRES), including the structural proteins core, E1 and E2. Assembly and release of particles composed of HCV structural proteins and containing the HCV-based RNA might then occur (Fig. 1C), and such particles might be infectious for appropriate target cells.

### Production of HCV bicistronic particles in BHK-WNV cells

**Figure 2:**
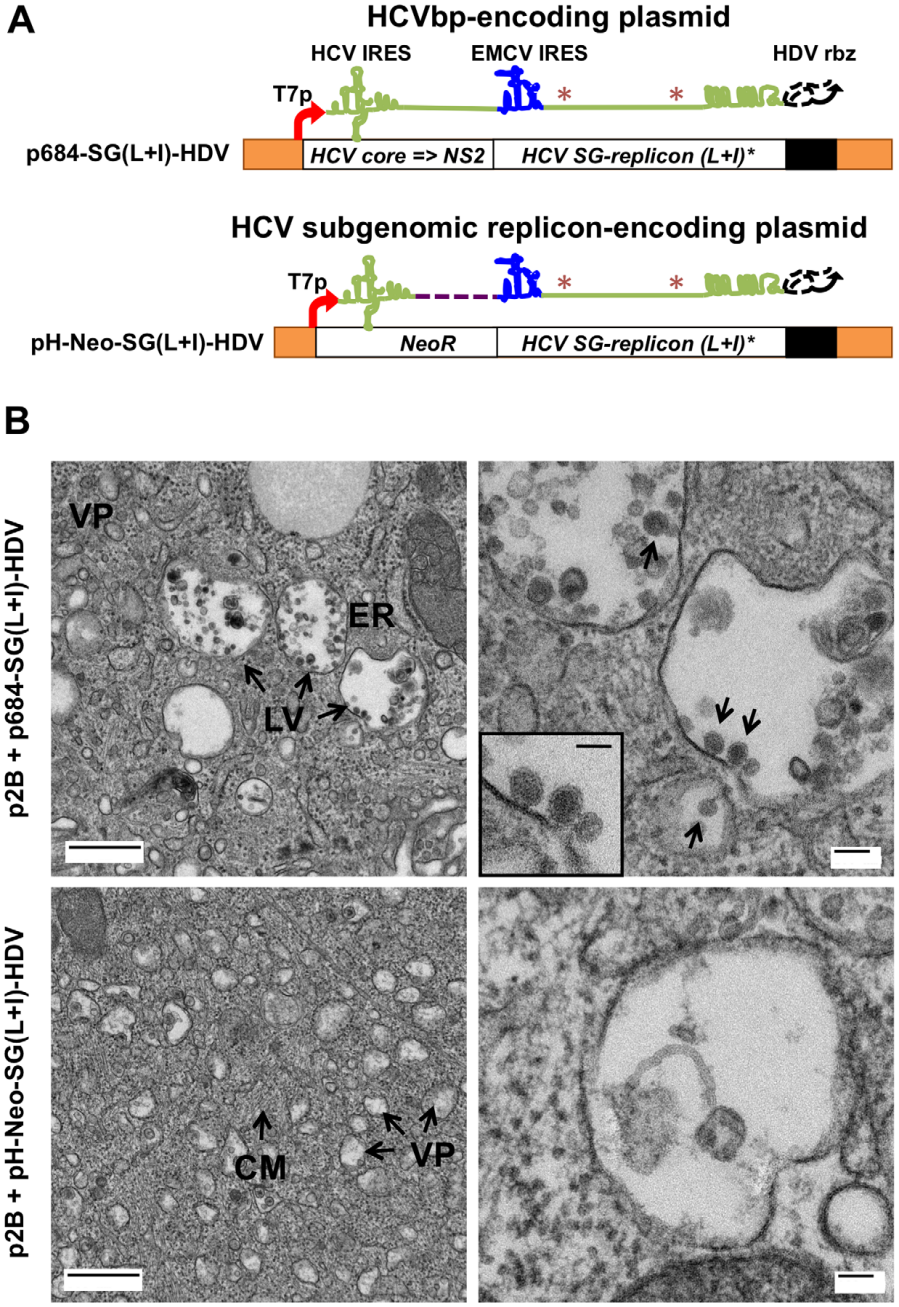
BHK-WNV cells assemble HCV bicistronic particles. (A) p684-SG(L+I)-HDV (top) is derived from pH-Neo-SG(L+I) plasmid (bottom), in which *NeoR* gene was replaced with HCV 5′-UTR to NS2 genes; a *HDVrbz* gene was introduced at the 3′ end of the HCV RNA of both constructs. It encodes a bicistronic full length HCV genome with cell-culture adaptive mutations (*). (B) BHK-WNV cells were transfected with the p2B system plus p684-SG(L+I)-HDV (top panels), or pH-Neo-SG(L+I)-HDV (bottom panels), and three days later subjected to TEM analyses: (Top panels) large vesicles (LV) filled with virus-like structures appeared (black arrow) next to dilated rough ER protrusions. At higher magnification: electron-dense, spherical, enveloped particles are budding into the lumen of LV (arrows). (Lower panels) BHK-WNV cells displayed extensive intracellular membrane rearrangements with numerous intracytoplasmic vesicles most likely representing the convoluted membranes (CM) and vesicle packets (VP) induced by WNV subgenomic replicon; these features are absent from parental cells (not shown). At higher magnification, these vesicles are mostly empty or contained non-specific cellular materials. Scale bars: left panels = 500 µm; right panels = 100 µm; inset, = 50 nm.

We first generated HCV bicistronic particles (HCVbp) using a plasmid encoding HCV 5′-UTR to NS2 sequence upstream of the encephalomyocarditis virus (EMCV) IRES of a Huh-7.5 cell-adapted HCV subgenomic replicon of subtype 1a [9], thereby yielding a bicistronic RNA (Fig. 2A, top) capable of replicating in Huh-7.5 cells. Co-transfection of BHK-WNV cells with this plasmid plus the p2B system resulted in the formation of large vesicles (not classical multi-vesicular bodies) filled with 50–60-nm particles in the vicinity of dilated rough ER protrusions and mitochondria, as observed by transmission electron microscopy (Fig. 2B, top panels). In contrast, BHK-WNV cells transfected with a control plasmid (HCV subgenomic replicon minus the HCV structural genes) displayed the extensive membrane rearrangements previously shown to be triggered by the WNV subgenomic replicon [25] (Fig. 2B, lower left panel), such as vesicle packets (site of WNV RNA replication) and convoluted membranes (site of WNV RNA translation and polyprotein processing); however the large vesicles containing particles were not observed (Fig. 2B, lower right panel). Immuno-gold electron microscopy analysis with anti-HCV E1 and anti-core antibodies revealed the presence of the corresponding HCV proteins within membrane rearrangements or large vesicles (Fig. S2) in BHK-WNV cells expressing the bicistronic HCV full length construct.

**Figure 3:**
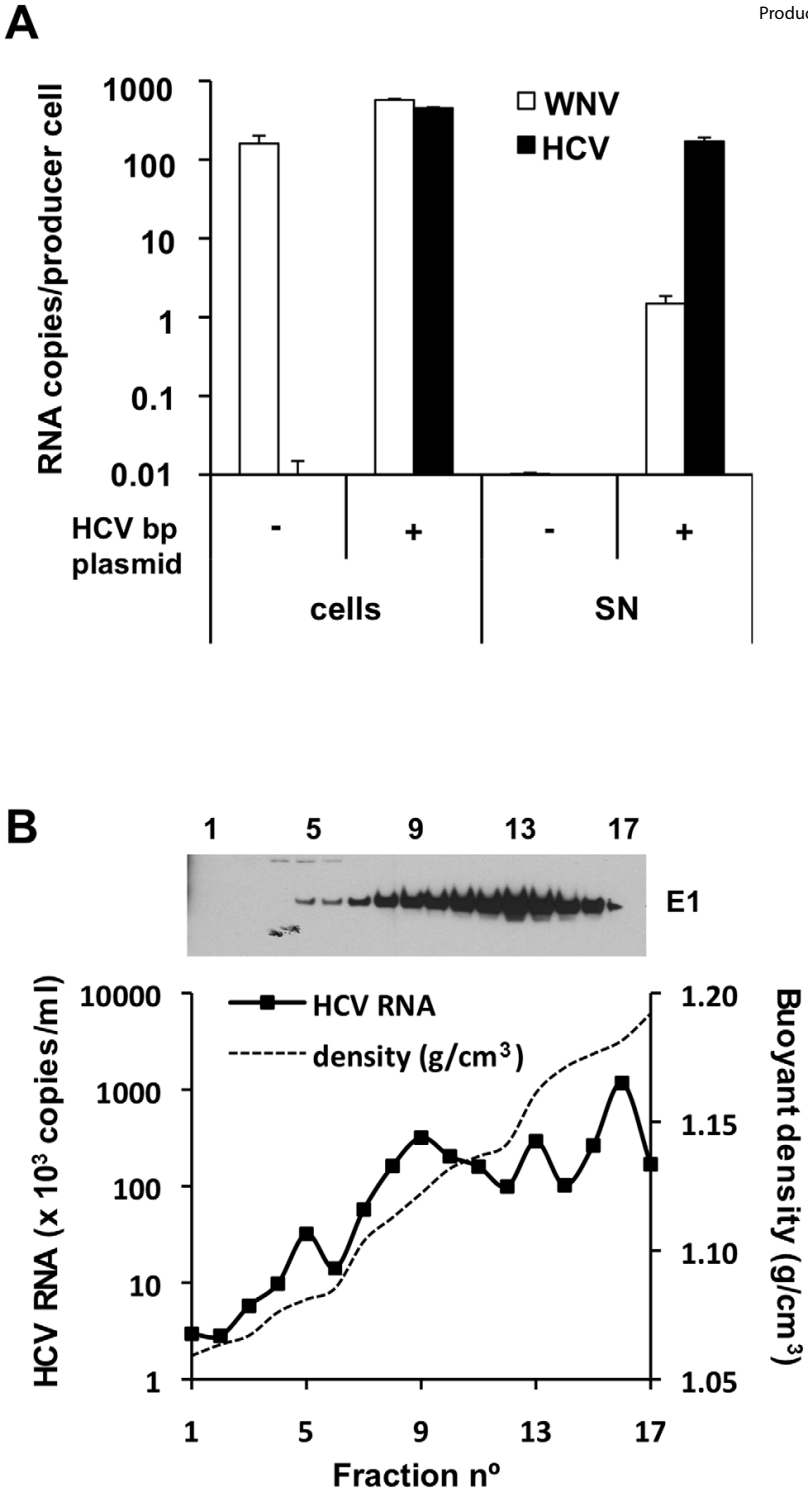
HCV RNA is preferentially associated with in HCV bicistronic particles released by BHK-WNV cells. (A) Three days after transfection of BHK-WNV cells with HCVbp-coding plasmid, RNA was extracted from cells and pellets of supernatants ultracentrifuged through sucrose cushion (SN); WNV and HCV RNA were each quantified by RT-qPCR; a similar protocol was used for control (untransfected) cells. The values on the Y-axis represent the amounts of cell- and SN-associated RNA extracted from an equivalent number of cells. (B) HCVbp released by BHK-WNV cells were analyzed on a 20–60% sucrose gradient; HCV E1 was detected by Western blot (top panel) and HCV RNA 5′-UTR measured by RT-Taqman PCR (bottom panel).

Fig. 3A shows quantitation of viral RNA (WNV and HCV) in BHK-WNV cells and the corresponding culture supernatants (SN) after their ultracentrifugation. As expected, the cells contained a large amount of WNV RNA generated by the WNV subgenomic replicon, independent of transfection with the HCVbp-encoding plasmid. In contrast, HCV RNA was observed only in cells expressing this plasmid, at levels comparable to the WNV RNA. Strikingly, this was accompanied by the appearance in the SN of a large amount of HCV RNA, which was highly enriched (approximately 100-fold) relative to the WNV RNA. Sucrose density gradient analysis of particulate material from the culture supernatant indicated that the HCV-based RNA migrated over a broad buoyant density range of 1.05 to 1.20 g/cm^3^ (Fig. 3B). The HCV E1 glycoprotein was detected across the gradient, as were the other structural proteins core and E2 (Fig. S3A, upper panels). These results suggest that the BHK-WNV cell system is capable of releasing particles composed of HCV structural proteins that are preferentially associated with the HCV-based RNA from which they were translated.

We determined that the harvested particles were not exosomes or cell debris, consistent with a requirement for maturation of HCV envelope proteins for particle release in our system (Text S1 and Fig S3A–C). We also excluded that the WNV RNA released upon transfection of the HCVbp plasmid in BHK-WNV cells (Fig. 3A) was associated with infectious particles. First, previous reports suggest a requirement for WNV core protein [26]. In addition, after the transfection of BHK-WNV cells with a plasmid encoding WNV structural proteins, the secreted particles (WNVrp) were infectious for Huh-7.5 cells (Fig. S3D), consistent with previous findings using other target cells [32]. However, incubation of Huh-7.5 cells with HCVbp did not yield any *Renilla* luciferase activity. Finally, BHK-WNV cells were treated with antiviral drugs for two weeks, which inhibited the WNV replicon (measured by the reduced expression of *Renilla* luciferase) but did not affect the release of HCV particles (Fig. S3E). It is therefore highly unlikely that HCV RNA replication is responsible for the production of HCV in this system (data on the mechanism will be presented elsewhere).

### Infectivity of HCVbp for Huh-7.5 cells

**Figure 4:**
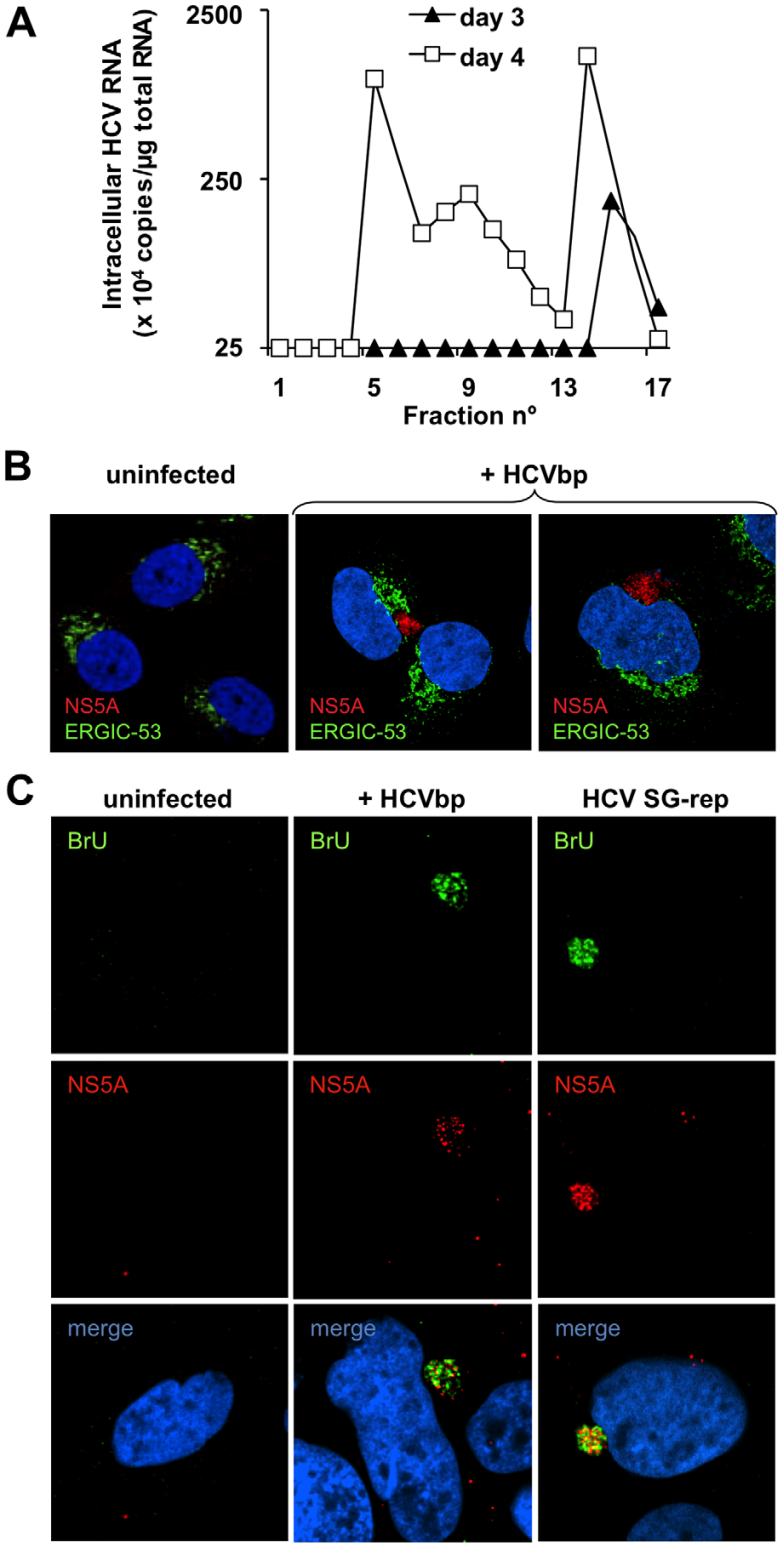
BHK-WNV cells produce HCV infectious in Huh-7.5 cells. (A) Huh-7.5 cells were incubated for 3 hr with aliquots of each fraction of the sucrose gradient shown in Fig. 3B, then transfected with plasmid encoding core to NS2 (which enhanced the readout); cellular contents in HCV 5′-UTR RNA were measured at the indicated time points by RT-TaqMan qPCR, with *in vitro* transcripts as standards (cf. Materials and Methods). (B) Huh-7.5 cells were incubated with HCVbp produced and harvested as described in Materials and Methods and, two days later, analyzed by confocal microscopy; NS5A was stained (red) with in-house rabbit polyclonal IgG; cells were counterstained with ERGIC-53 (green) and DAPI (blue). (C) Two days after infection with HCVbp, Huh-7.5 cells were incubated with BrUTP and NS5A antibody. Huh-7.5 cells with an established HCV subgenomic replicon were used as a positive control.

Several criteria were examined to test the infectivity of the HCVbp in Huh-7.5 cells. First, we used RT-qPCR for the 5′-UTR to test individual fractions from the sucrose density gradient in Fig. 3B for their ability to induce HCV RNA replication. As shown in Fig. 4A, the amounts of HCV RNA in target cells at day 3 post-infection were negligible for nearly all fractions, and increased substantially by day 4. As previously reported for HCVcc [23], [33], we observed that the infectivity of HCVbp was spread over a broad range of buoyant densities, and that it did not directly correlate with the detected amounts of viral RNA. The peak of infectivity generally ranged between 1.08–1.13 g/cm^3^ (Fig. 4A), which corresponded to a low peak of HCV RNA (Fig. 3B). Infectious titers of HCVbp in the supernatants of BHK-WNV cells were measured in Huh-7.5 cells. TCID_50_ were between 0.6×10^4^ units/ml at day 3 and 2.5×10^5^ units/ml at day 4 (cf. Text S1), consistent with data presented in Fig. 4A. Such viral titers are about one log lower than with the JFH-1 strain [11], [12] and genotype 2a chimera [13] after a two-day incubation in permissive cell lines, but at least 10-fold higher than with HCVcc obtained with a cell culture-adapted strain of genotype 1 [23].

The relatively low buoyant density of most infectious particles could relate to their association with lipids, since lipid droplets were detected in the vicinity of non-structural proteins in BHK-WNV cells expressing the HCVbp-4cys construct, encoding a tetracysteine tag within NS5A (Fig. S4A). As BHK-21 cells express functional LDL receptor [34], another non-exclusive possibility is that HCV particles interacted with lipoproteins from the culture medium. Incubating HCVbp with (up to 0.15 µg/ml) human VLDL, LDL or HDL *in vitro* prior to Huh-7.5 cells enhanced the amount of viral RNA accumulating in target cells up to 5-fold (not shown), which would be consistent with a specific interaction of lipoproteins with pre-assembled HCVbp, as previously reported for HCV-like particles (HCV-LPs) [35], lentiviral particles pseudo-typed with HCV envelope proteins (HCVpp) and HCVcc [36].

We also analyzed HCVbp-induced synthesis of HCV proteins and RNA in Huh-7.5 target cells by laser-scanning confocal microscopy. Based on staining with a polyclonal antibody against NS5A (Fig. 4B; specificity of antibody validated in Fig. S4C), NS5A-positive patches were detected in the cytoplasm of Huh-7.5 cells infected with HCVbp for two days (center and right panels), but not in uninfected cells (left panel). Albeit in close proximity with ERGIC53, these patches did not co-localize with this lectin that transports glycoproteins from the ER to the Golgi apparatus, suggesting that NS5A was not associated with a ‘classic’ membrane compartment. We also examined HCV RNA replication in Huh-7.5 cells incubated with HCVbp; after several hours, the cells were treated with actinomycin D to block RNA polymerase II-dependent nuclear transcription, then loaded with 5-bromo-UTP, a nucleotide analog that is incorporated into elongating RNA. Staining of HCVbp-infected cells with anti-bromo-uridine (BrU) and NS5A antibodies resulted in the detection of both signals in a cytoplasmic subcompartment of Huh-7.5 cells incubated with HCVbp (Fig. 4C, center panels). This staining pattern was very similar to that observed in positive control cells, i.e. Huh-7.5 cells bearing an HCV subgenomic replicon (Fig. 4C, right panels), but not observed in the uninfected negative control cells (Fig. 4C, left panels). This result presumably reflects the local incorporation of BrU into replicating HCV-based RNA, as has been shown for flaviviruses [37]. Consistent results were obtained with live cells infected with particles encoding a tetra-cysteine tag in NS5A (Fig. S4D).

Treatment of cells with viral inhibitors (interferon α or β plus ribavirin) prior to their inoculation with HCVbp inhibited the accumulation of viral RNA by ∼10-fold (not shown). The sensitivity of HCV replication to these agents [38] suggests that HCVbp-mediated increase in HCV RNA reflects the activity of the introduced subgenomic replicon. The pre-incubation of HCVbp with serum from an HCV-cured patient (without circulating HCV RNA by PCR) decreased the amount of intracellular RNA (Fig. S4B) detected by RT-qPCR, compared to that with normal/naive human serum, suggesting the existence of a specific interaction of HCVbp with the immune serum (presumably IgG) interfering with their infectivity.

### Receptors involved in HCVbp entry

**Figure 5:**
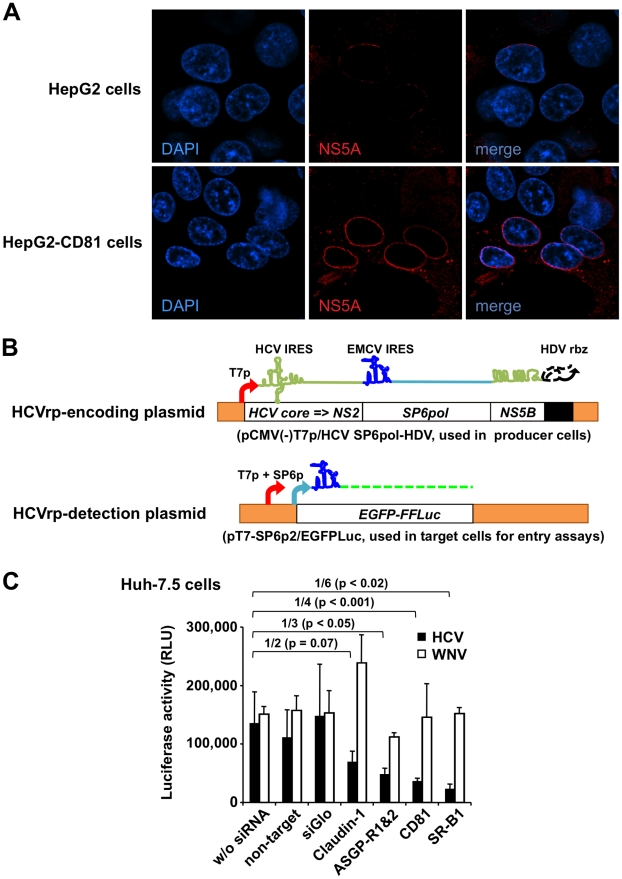
Study of entry of BHK-WNV cell-produced HCV particles. (A) Parental HepG2 and HepG2-CD81 cells were incubated with HCVbp and NS5A expression was analyzed two days later by laser scanning confocal microscopy. (B) pCMV(-)T7p/HCV SP6pol-HDV plasmid was used to produce HCVrp that encapsidate a bicistronic reporter RNA encoding HCV core to NS2 and SP6 Pol, and ending with HCV NS5B C-terminus to 3′-UTR RNA ( = kissing loops). To detect incoming-*SP6pol* RNA following HCVrp entry, target cells were transfected with a reporter plasmid (pT7-SP6p2/EGFPLuc); its co-transfection with the p2B system enhanced the cytoplasmic transcription of the reporter plasmid triggered by incoming *SP6pol* RNA in target cells; a treatment with actinomycin D decreased the background signal. (C) HCVrp and WNVrp were incubated with siRNA-treated Huh-7.5 cells, as indicated. HCV (filled bars) or WNV (open bars) incoming signals into target cells were measured using *Firefly* or *Renilla* luciferase assay, respectively. Error bars represent the SD of triplicate experimental points; these results are representative of n = 5 independent experiments.

The CD81 tetraspanin has been implicated as an important receptor for HCV entry [39]. Albeit of human hepatic origin, the HepG2 cell line lacks CD81 and is poorly permissive for HCV entry but can be rendered permissive by CD81 expression, as previously shown by infection with HCVpp [40] or HCVcc [41]. We found that stable transduction of these cells with a recombinant lentivirus encoding human CD81 resulted in its surface expression (Fig. S5A); it enhanced the NS5A signal triggered by the incubation of HepG2 cells with HCVbp (Fig. 5A; compare right and left panels).

For more quantitative analyses, we devised a variation of the system involving the production of HCV reporter particles (HCVrp). To this end, the fragment encoding NS3 up to the last third of HCV NS5B in the HCVbp construct was replaced with one encoding the ORF of bacteriophage SP6 RNA polymerase (SP6 Pol; Fig. 5B, top). After HCVrp entry into target cells, these cells were co-transfected with the p2B system plus a plasmid encoding EGFP fused with *Firefly* luciferase, linked to the T7 and SP6 promoters and an EMCV IRES (Fig. 5B, bottom), and were treated with actinomycin D to decrease the background reporter gene expression in the absence of incoming SP6 Pol-encoding RNA, which triggers reporter gene expression in a dose-dependent manner, independent of most post-entry processes. As predicted, parental BHK failed to release infectious HCVrp (Fig. S5B). Although EGFP expression was also observed, only luciferase activity is reported. We tested the dependence of HCVrp entry (cf. Text S1 and Table S1) on surface molecules previously implicated as essential entry receptors in target cells (Fig. 5C). Inhibition of the *Firefly* luciferase signal generated by HCVrp entry occurred when Huh-7.5 cells were pretreated with siRNA pools targeting several HCV candidate receptor molecules: SR-B1 [42], CD81 [39], ASGP-R subunits 1 and 2 [43], and to a lesser extent claudin-1 [44] (Fig. 5C, filled bars). The same siRNA treatments had little effect on entry of WNVrp (generated by transfecting BHK-WNV cells with a plasmid encoding WNV structural proteins), as measured by the *Renilla* luciferase activities encoded by the WNV subgenomic replicon packaged into WNVrp (Fig. 5C, open bars). SR-B1 siRNA was the most effective at blocking both the protein expression (Fig. S5C) and HCVrp entry (Fig. 5C). Consistently, pre-incubation of Huh-7.5 cells with antibodies against CD81 and SR-B1 significantly inhibited HCVrp entry signal (Fig. S5D). The interaction of HCV E2 hypervariable region 1 (HVR-1) interaction with SR-B1 is critical for infection [42] and *in vivo* infection has previously been neutralized by an antiserum against HVR-1 [45]. Preliminary data (reagent was made available in very limited quantity) shows that incubation of HCVrp with these anti-HVR-1 antibodies also inhibited their entry into Huh-7.5 cells (Fig. S5E).

### Production of JFH-1 strain-based HCV by BHK-WNV cells

**Figure 6:**
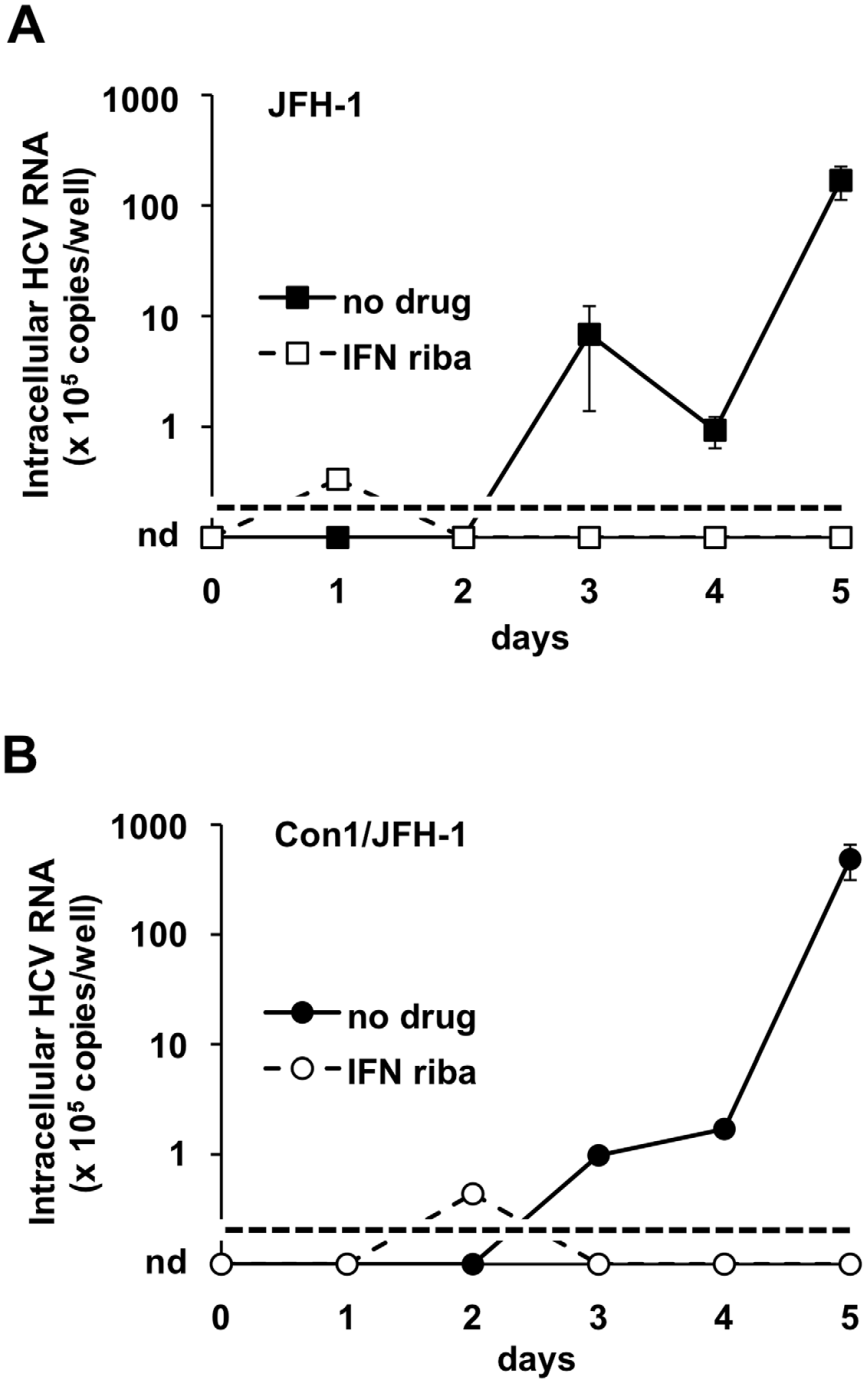
JFH-1-based HCV produced in BHK-WNV cells infect Huh-7.5 cells. Huh-7.5 cells were seeded one day before the experiment and treated with (open symbols, dashed lines) or without (closed symbols) leukocyte interferon (100 IU/ml) plus ribavirin (400 µM). JFH-1 (A) and Con1-JFH1 (B) HCV particles produced in BHK-WNV cells were incubated with the Huh-7.5 cells for 2 hrs; after several washes, the cells were split and an equivalent amount of cells were either directly harvested (day 0) or seeded onto collagen-I-coated 24-well plates (and interferon plus ribavirin treatment was re-introduced where applies) and further cultured for the indicated times. Cells were then harvested and HCV 5′-UTR RNA was subject to RT-qPCR analysis, as in (Fig. 4A). Errors bars represent SD of 4 measurements; the limit of detection in this assay is indicated by a dotted line; nd = undetermined values (i.e. <10^5^ HCV RNA copies/well).

We also tested the possibility of producing infectious particles based on the ability of the JFH-1 strain to infect Huh-7.5 cells [11]. Plasmids encoding the genomic RNA of JFH-1 [11] or a Con1-JFH1 (1b-2a) chimera [18] under a T7 promoter were transfected into BHK-WNV producer cells and HCV particles were harvested, then incubated with Huh-7.5 cells. Fig. 6A–B shows the detection of viral RNA in the target cells for both constructs. Starting at day 3, increasing RNA amounts were measured, whereas in cells treated with interferon plus ribavirin no such increase was detected (Fig. 6A–B).

### BHK-WNV cells produce authentic infectious HCV

**Figure 7:**
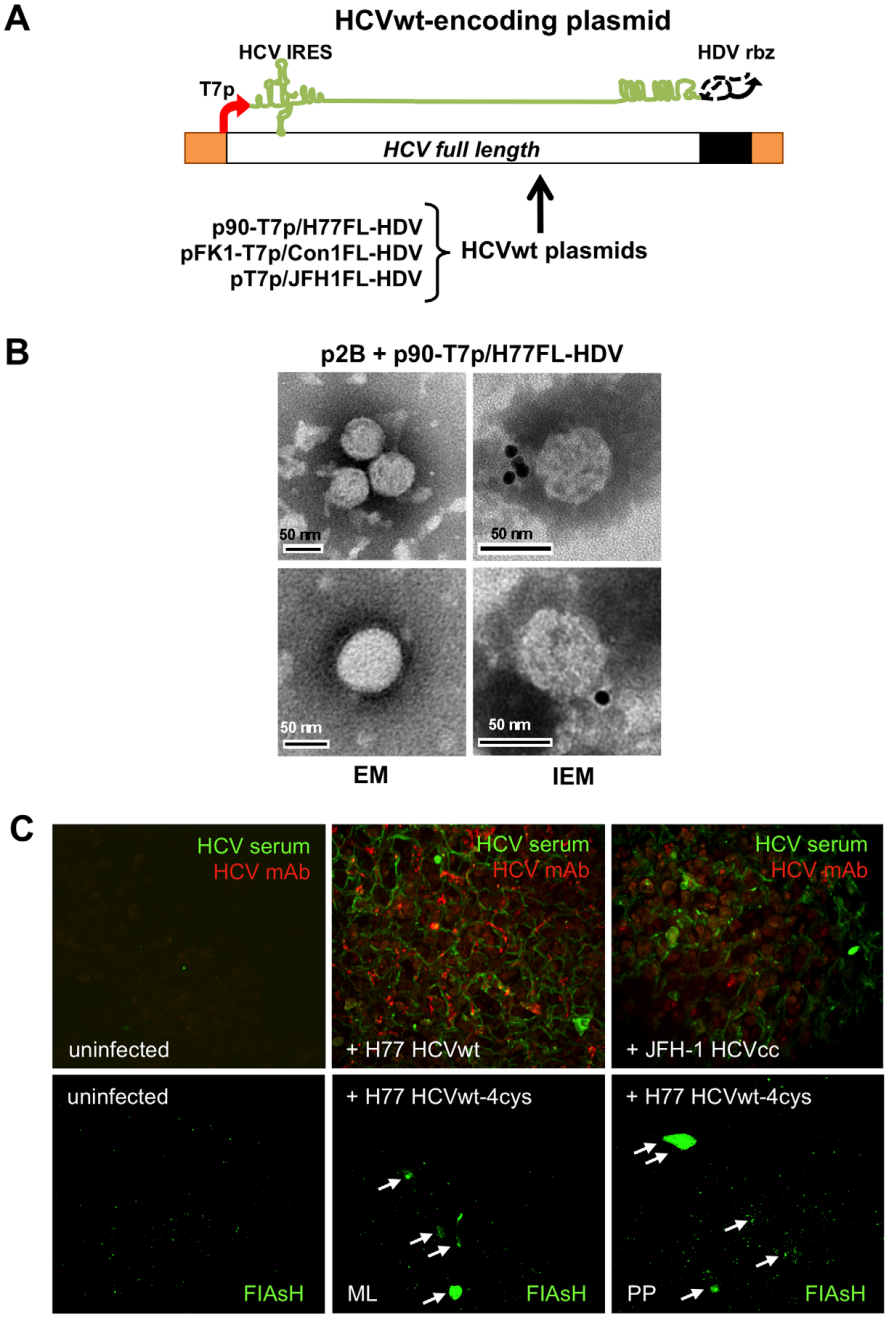
Wild-type HCV produced in BHK-WNV cells infect human liver slices. (A) Transfection of p90-T7p/H77FL-HDV in BHK-WNV cells yielded authentic HCV particles (HCVwt) that encapsidate a full-length, wild-type genome of H77 strain. (B) HCVwt particles released by BHK-WNV cells were analyzed by ultracentrifugation on a sucrose gradient; fractions with buoyant densities of 1.08–1.13 g/cm^3^ were pooled and observed by negative staining (EM) or immuno-gold electron microscopy (IEM, using serum from HCV-cured patient). (C) (Upper panels) Human liver slices were uninfected (left), or infected with BHK-WNV-produced HCVwt (H77, center), or HCVcc produced in Huh-7.5 cells (JFH-1, right), then cultured for 8 days. Co-incubation with anti-HCV antibodies (cf. Materials and Methods) resulted in specific staining observed over a thickness of 60–70 µm with a multifocal confocal microscope. (Lower panels) Human liver slices were infected with BHK-WNV-derived HCVwt-4cys for 6 days, then stained with FIAsH and observed over a thickness of ∼150 µm with a multiphoton confocal microscope; ML = mediolobular area; PP = periportal space; white arrows indicate positive cells.

As an additional variation of this HCV expression approach, we tested the possibility that BHK-WNV cells could produce authentic infectious HCV particles. We co-transfected BHK-WNV cells with the p2B system and a plasmid encoding a full-length genomic RNA with the consensus sequence of a strain of genotype 1a (H77, Fig. 7A), which has been shown to be infectious in chimpanzees [46]. The ‘wild type’ particles (HCVwt) released into the supernatants were harvested by ultracentrifugation and analyzed by sucrose density gradient centrifugation. In fractions with buoyant densities of 1.08–1.13 g/cm^3^, spherical particles of 50–60 nm in diameter were observed by negative staining electron microscopy; these were not observed with corresponding fractions from control BHK-WNV cells. Some of these particles were positive by immuno-gold electron microscopy, indicating their recognition by immunoglobulins from an HCV-cured patient (Fig. 7B).

As HCV isolates from patients are poorly infectious in Huh-7 cells [6], the infectivity of HCVwt was tested in liver slices from non-infected patients (negative for HCV, HBV and HIV). Like primary human hepatocytes [47], liver slices can be infected *ex vivo* with HCVcc (unpublished). The liver slices presumably better reflect the real situation than cell lines do, as both the architecture and cell type diversity of the liver are maintained in their original configuration. After incubation of liver slices with BHK-WNV cell-produced HCVwt (H77 strain) [46] or Huh-7.5 cell-produced HCVcc (JFH-1 strain) [11], specific staining by anti-HCV antibodies was analyzed by multifocal confocal microscopy; after a few days, the signal appeared within the slices at various locations of a few lobules, and increased up to 6–10 days. Fig. 7C (upper panels) shows data obtained at day 8; positive staining by both serum from HCV-infected and monoclonal antibodies against structural proteins was observed (middle panel) within two to five lobules of HCVwt-infected slices (area >1 cm^2^). The results were similar to those obtained after infection with HCVcc (right panel), and specificity was verified by the undetectable staining in uninfected liver slices (left panel). Infection was detected in clusters of cells within a few lobules, consistent with a recent report showing that HCV infection of the liver involves a limited number of hepatocytes [48]. Results varied in shape and intensity with liver donor, but the specificity of the detected infection signal was further confirmed by additional analyses with control antibodies (Fig. S6).

Similar results were obtained with HCVwt-4cys, encoding a tetracysteine tag within its non-structural gene NS5A, as previously validated in Huh-7.5 cells (cf. Text S1). Six to eight days after their infection with HCVwt-4cys, liver slices were incubated with a permeable biarsenical dye and observed with a two-photon confocal microscope. Specific staining was detected predominantly in a few periportal spaces, and also in mediolobular areas (Fig. 7C, lower middle and right panels) of HCVwt-4cys-incubated slices. In spite of a high background that reduces the sensitivity of detection with this technology, the appearance of small clusters of positive signals (generated in live cells) is consistent with the local synthesis of HCV non-structural proteins in human liver slices after their *ex vivo* infection with HCVwt-4cys produced in BHK-WNV cells.

### HCV produced in BHK-WNV cells infect HepG2-CD81 cells

**Figure 8:**
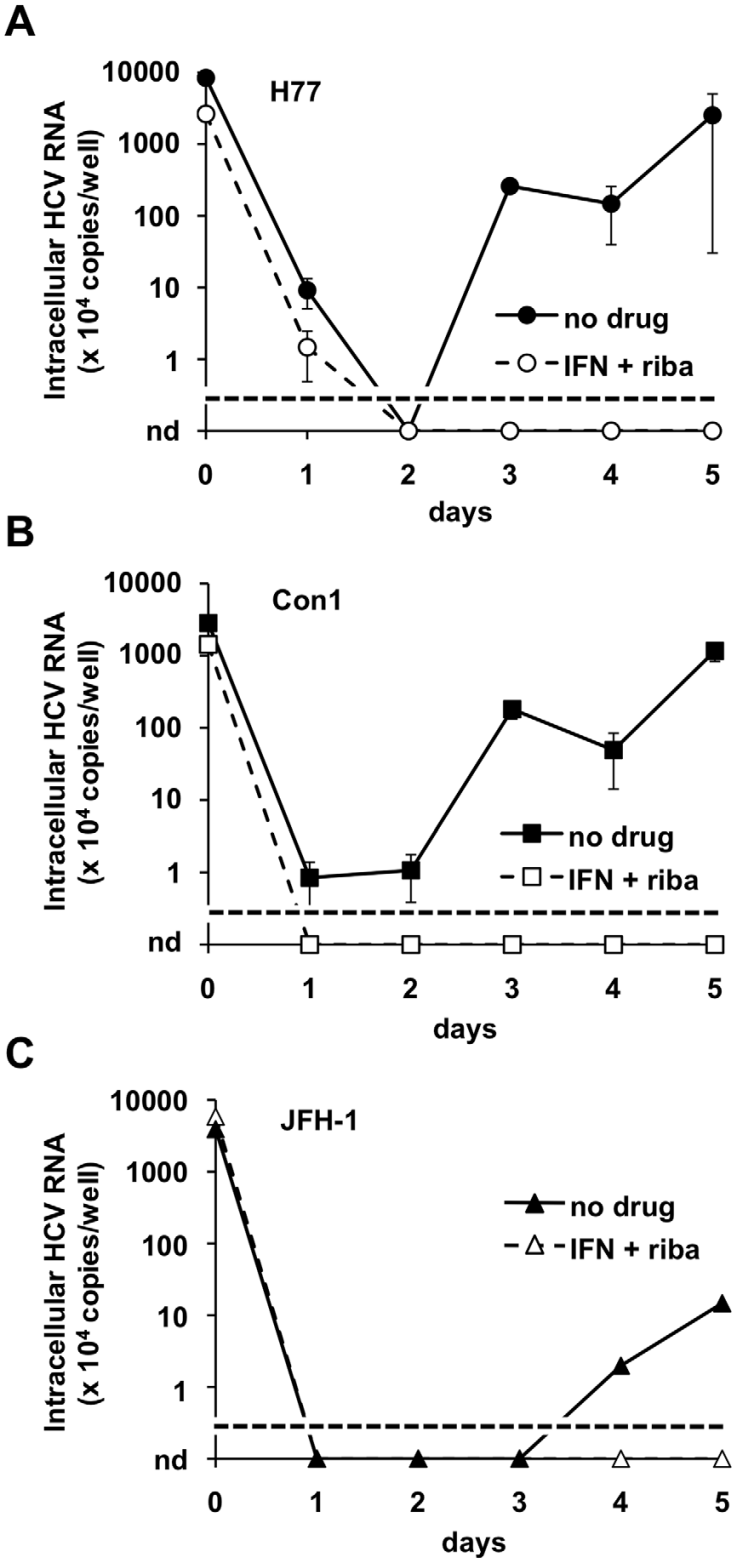
Wild-type HCV produced in BHK-WNV cells infect HepG2-CD81 cells. HepG2-CD81 cells were seeded and treated one day prior to the experiment with (open symbols, dashed lines) or without (closed symbols) leukocyte interferon (100 IU/ml) plus ribavirin (400 µM). HCV particles were produced with the wild type genomes of H77 (A), Con1 (B) and JFH-1 (C) strains (of genotypes 1a, 1b and 2a, respectively) and harvested, as described in Materials and Methods. The cells were incubated with particles for 3 hrs, then washed, split and seeded onto collagen-I-coated plates (IFN+ribavirin treatment was re-introduced where applies). At the indicated times, cells were harvested and HCV genomic RNA was subject to RT-qPCR analysis, as in Fig. 4D. Results representative of 3 independent experiments performed in duplicates; the limit of detection in this assay is indicated by a dotted line; nd = undetermined; errors bars represent SD of 4 measurements.

As HCV isolates from patients are poorly replicating in Huh-7 cells [6], [9], [22] and access to naïve human liver slices of good quality is limited, we tested the possibility that HCVwt could infect HepG2-CD81 cells, which have been previously reported to support replication of patient isolates [6]. To some extent these cells support HCVbp replication (Fig. 5A). The incubation of HepG2-CD81 cells with HCVwt (produced in BHK-WNV cells) of subtypes 1a, 1b, and to a lesser extent 2a (or 1b/2a chimera; not shown), resulted in high readings starting at day 0 (Fig. 8A–C). Although the detected amounts of HCV RNA sharply decreased during the first 24–48 hr, which could relate to some non-productive binding/uptake, it raised again afterward; the later increase was abolished by a treatment with interferon and ribavirin added to the cells both prior to and after infection (cf. results in Huh-7.5 cells). Incubation of HepG2-CD81 cells with HCVwt of subtypes 1a resulted in more intracellular accumulation of HCV RNA than what was measured after their incubation with HCVbp of the same genotype (not shown); one possible interpretation is that Huh-7.5 cell-adaptive mutations were detrimental to HCVbp replication in HepG2-CD81 cells, similar to what has previously been reported in the liver, *in vivo* [20].

## Discussion

Producing large amounts of infectious HCV virions in cultured cells has been difficult, especially for the most prevalent and clinically problematic genotype 1, which in part relates to its poor ability to replicate *in vitro* and the subsequent appearance of cell culture-adaptive mutations interfering with its propagation and infectivity. Here, we produced HCV particles of genotype 1 containing a genome previously shown to be highly infectious *in vivo* [7], [46]. Their ability to infect human liver slices demonstrates the biological relevance of the particles produced in this *in vitro* system. Two major features underlie independence from HCV replication, which avoided adaptive mutations typically associated with HCV propagation in cell culture: first, the unique and robust strategy for producing HCV genomes in the cytoplasm independent of HCV replication, and second, the WNV subgenomic replicon that created an appropriate cellular environment for HCV RNA translation as well as particle assembly and release.

HCV particle formation likely took place within membrane rearrangements derived from those induced by the WNV subgenomic replicon, as suggested by immuno-gold electron microscopy results. We also observed that the release of HCV particles by BHK cells was enhanced by lineage I WNV [49] and serotype-2 dengue virus [50] subgenomic replicons, but not by one of Semliki Forest virus [51], an alphavirus belonging to the *Togaviridæ* family (Fig. S1B). This indicates that, beyond similarities in genomic organizations and sequences [31], the increased production of infectious HCV could result from common functional properties conserved amongst members of the *Flaviviridæ* family rather than strict sequence specificity of the proteins encoded by the flavivirus subgenomic replicons. In BHK-WNV cells, this possibility is further substantiated by the lack of correlation between HCV production and translation of the WNV subgenomic replicon, upon inhibition of the latter's activity. The replication of flaviviruses and HCV induce similar membrane rearrangements in the cytoplasm of infected cells [24], [25], and our data confirmed that flaviviruses also infect hepatocytes [28], [52]. In Huh-7.5 cells, cholesterol metabolism has been implicated in HCV replication [53] and lipid droplets in its assembly [54]. Likewise, WNV replication involves cholesterol metabolism [55] and, for dengue virus particle formation, the interaction of the viral core with lipid droplets [56]. As the mechanisms involved in the production of HCV by hepatocytes are still debated, these similar features perhaps underlie part of the BHK-WNV cell permissiveness for HCV particle formation.

The correlation between reversal of membrane rearrangements and loss of HCV particles production (not shown) suggests that these rearrangements, and perhaps related cellular changes (e.g. cholesterol metabolism and lipid droplet formation), are playing a major role in the permissiveness of BHK-WNV cells. However, the down or up regulation of other cellular factors could be involved as well. Thus, several intracellular mechanisms involved in innate immunity interfere with flavivirus propagation [e.g. 57]–[59], and knockdown of interferon stimulating mechanisms or signaling pathways enhance WNV [58], [59] and HCV [60] productions in cell culture; WNV [61] and HCV [62] proteins have been shown to directly target such pathways. Here we cannot exclude that such a mechanism took place prior to or upon expression of HCV genes. However, the introduction of a BHK cell-adapted WNV subgenomic replicon into naïve BHK-21 cells rendered them rapidly permissive for the production of WNV, whereas that of HCV appeared after many more passages (not shown). One possible interpretation is that co-evolution of WNV subgenomic replicon and BHK cells under antibiotic selection led to the regulation of additional cellular factors, probably involved in fine tuning WNV replication and/or translation, but absolutely required for the production of infectious HCV. This prompted us to identify such cellular factors in BHK-WNV cells and test their relevance with the JFH-1 strain/Huh-7.5 cells paradigm, the results of which will be presented elsewhere.

The entry assay with particles produced in BHK-WNV cells (HCVrp) requires only the delivery of the associated RNA molecule into the cytoplasm of the target cell where it can be translated at sufficient levels to trigger the dual bacteriophage RNA polymerase amplification system. Thus, the target cell needs to be permissive only for viral entry, and possibly a limited number of post-entry steps (e.g. RNA uncoating). Most importantly, the non-involvement of RNA replication for the signal readout allows assessment of the entry permissiveness of diverse cell types, independent of their ability to support HCV replication. This represents a significant advantage over the HCVcc system that also relies on viral spreading to amplify the read out signal, and the involvement of only HCV structural proteins and RNA clearly distinguishes this system from HCVpp, which is based on non-HCV protein and nucleic acid platform.

We had previously observed that both ASGP-R subunits were required for internalization of HCV materials into hepatocellular carcinoma as well as non-target cells [43]. Here we show that these subunits are involved in delivering HCV reporter RNA into HCV-permissive hepatic cells. It is not yet known whether the role of ASGP-R in HCV uptake relates to incomplete maturation of E1 and/or E2 carbohydrate residues, as previously observed [63], [64], or involves another mechanism [65], [66]. HCV has been reported to enter cultured cells *via* clathrin-coated pits [67]–[69], and ASGP-R internalization follows the same path [66], [70]. Yet, ASGP-R can be targeted to various intracellular compartments including ER [43], which leaves open the possibility that this receptor plays a role at an early as well as a late step of the HCV entry process and RNA delivery.

As inter-genotypic differences and cell-adaptive mutations could affect viral production in hepatic cells, the BHK-WNV paradigm provides an alternative model to produce wild type virus for *in vivo* or *ex vivo* studies without the concern that adaptive mutations develop. It could also present major advantages for deciphering mechanisms of viral translation, assembly, release and entry, including involvement of non-structural genes in viral production independent of their role in replication.

## Materials and Methods

### Cell cultures

BHK-21 cells were grown in E-MEM supplemented with 10% fetal bovine serum (FBS; HyClone), GlutaMax-I (Invitrogen); BHK cells harboring WNV lineage II SG-replicon encoding *Renilla* luciferase, BHK WNIIrep-REN cells [32], herein simply called BHK-WNV cells, were propagated in D-MEM supplemented with 10% FBS, GlutaMax-I and 5 µg/ml blasticidin (Invitrogen). Huh-7.5 cells and Huh-7.5 cells harboring HCV SG-replicon of 1a genotype (H77) with mutations in NS3 and NS5A (Huh-7.5-SG 1a rep) were maintained as described [9], [10]. HepG2 cells were grown in E-MEM supplemented with 10% FBS, GlutaMax-I and non-essential amino acid mix. Cells were cultured in an incubator with a 95% air/5% CO_2_ atmosphere saturated in humidity.

### Plasmid constructs

A new system of plasmids (p2B) was designed to amplify the cytoplasmic transcription of plasmids in which the gene of interest is under the control of a DNA-dependent RNA polymerase (DdRp)'s cognate promoter; this system consists of a set of two plasmids generating T7 polymerase (T7 Pol): **1)** **pCR-T7p/SP6pol** in which bacteriophage SP6 DdRp (*SP6pol*) gene was cloned into pCR2.1 plasmid (Invitrogen) in frame with the second ATG start codon of EMCV IRES under the control of T7 promoter; **2)** **pSL-SP6p/T7pol** in which bacteriophage T7 DdRp (*T7pol*) gene was cloned into pSL1180 plasmid (Clontech) in frame with the second ATG start codon of EMCV IRES under the control of SP6 promoter. This p2B system was used for all T7 Pol promoter-driven HCV coding plasmids, in which a sequence coding for an HDV antigenomic ribozyme [71] was added at their C termini. p90 HCVconFLlongpU encoding the FL genome of infectious H77 strain [46], or, pH-Neo-SG(L+I) encoding a subgenomic replicon of the same strain with cell-culture adaptive mutations [9] were used as templates to construct all HCV coding plasmids of genotype 1a. HCVbp was produced from **p684-SG(L+I)-HDV** plasmid, in which the neomycin resistance gene of **pH-Neo-SG(L+I)-HDV**, i.e. pH-Neo-SG(L+I) encoding an hepatitis delta virus antisense ribozyme (HDV rbz) after the HCV 3′-end, was replaced with HCV 5′-UTR to NS2 coding sequence. An *HDVrbz* gene was introduced at the 3′-end of p90HCVconFLlongpU to create **p90-T7p/H77FL-HDV** plasmid that will produce HCVwt, i.e. virus particles containing the full-length, consensus sequence of H77 strain. HCVbp-4cys and HCVwt-4cys were obtained using modified **p684-SG(L+I)-HDV** and **p90-T7p/H77FL-HDV** plasmids, in which a tetracysteine tag-encoding sequence [72] had been inserted within the NS5A gene. HCVrp was produced from **pCMV(-)T7p/HCV-SP6pol-HDV** plasmid that encodes HCV 5′-UTR and structural genes followed by those of *SP6pol* (entry signal) gene in frame with EMCV IRES and a sequence encoding carboxy-terminus of HCV NS5B (kissing loops) [73] and 3′-UTR. To detect incoming-SP6pol RNA upon HCVrp entry into target cells, **pT7-SP6p2/EGFPLuc** reporter plasmid was made. This plasmid was derived from pEGFPLuc plasmid (Clontech) in which EMCV-IRES-EGFPLuc expression is under the control of both bacteriophage T7 Pol and SP6 Pol cognate promoters in tandem. This construct lacks eukaryotic promoter and therefore is responsive either to T7 Pol, SP6 Pol, or both; it was found responding to either incoming DdRp, be it in the form of protein or DdRp encoding RNA (not shown). Two additional constructs, pHCVp7 and pHCVcore-NS2 are pcDNA3.1(+)-based plasmids (Invitrogen), respectively encoding HCV 1a structural genes (core, E1, E2, p7) and HCV 1a structural genes plus NS2. pIRES1hyg-WNV [32] encodes WNV structural genes (core, prM and E). These three plasmids are under CMV early promoter (not shown). pJFH1 [11], pFK1-Con1 (9605Con1) [7] and pFK-JFH1Con1C-842 [18] are plasmids encoding from a T7 Pol promoter the genomic RNA of, respectively, the JFH-1 strain (genotype 2a), the Con1 strain (genotype 1b) and a Con1-JFH1 chimera (1b/2a). A DNA fragment encoding an HDV rbz was inserted at the 3′-end of the HCV RNA coding region of each plasmid.

### Antibodies and live-cell staining

Anti-E2 monoclonal antibodies (ALP98 and AP33) [74] and anti-E1 (A4) monoclonal antibody were used for Western blot analysis, and rabbit polyclonal antibody against HVR1 of E2 [45] for inhibition of HCVrp entry. Anti-NS5A rabbit polyclonal antibody (in-house) was used for confocal microscopy analysis. To produce rabbit antibody against NS5A of genotype 1a, 48-amino-acid peptide: NH_2_-AEEDEREVSVPAEILRKSRRFARALPVWARPDYNPPLVETWKKPDYEP-COOH, corresponding to position 2261–2308 of the H77 strain was synthesized by Peptide Synthesis and Analysis Laboratory (RTB/NIAID/NIH); a cysteine residue was introduced at the amino-terminus and the peptide was coupled to KLH. Two rabbits were immunized from which two sera were harvested; both IgGs were peptide affinity-purified. Sequence of the peptide is almost identical (but amino acids 22, 25, 43 and 46) to that of Con1 (genotype 1b). Monoclonal antibody against HCV core protein (clone C7-50; Thermo Scientific) was used to analyze Huh-7.5-produced JFH-1 (HCVcc) infection by confocal microscopy. Antibodies against HCV candidate receptors and cellular proteins are as follow: anti-CD81 mAb (JS-81, BD Biosciences); anti-SR-BI rabbit polyclonal antibody (Novus Biologicals); anti-ASGPR-1 mAb (clone 8D7, Santa Cruz Biotechnology); anti-claudin mAb (Invitrogen); anti-Hsp70 (BD Biosciences); anti-ERGIC-53 (Alexis Biochemicals) and anti-BrdU (Invitrogen). FIAsH- and ReAsH-EDT2 labeling reagents were obtained from Molecular Probes (Invitrogen). For flow cytometry and immunofluorescence (confocal microscopy) analysis, the secondary antibodies used were Alexa Fluor 488-, 594-, or 635-conjugated goat anti-mouse and anti-human antibodies, and Alexa Fluor 594-, 635-, or 680-conjugated goat anti-rabbit antibodies from Molecular Probes (Invitrogen).

### Production of HCV particles

One day before transfection, BHK-WNV cells were seeded at a density of 6×10^6^ cells per 162-cm^2^ flask. Plasmids encoding HCV sequence under the control of bacteriophage T7 promoter (or CMV early promoter where specified) were transfected using Lipofectamine LTX and Plus reagent according to the manufacturer's protocol (Invitrogen). Culture medium after transfection was D-MEM supplemented with 10% FBS, 1% non-essential amino acid mix, GlutaMax-I, 25 mM Hepes; cells were incubated at 37°C. One or two days later, 2.5 to 3.7 g/L sodium bicarbonate was added (to prevent further acidification of the medium), and culture medium were harvested at day 3, centrifuged at 30,000× *g* for 30 min at 4°C to remove cell debris, then clarified supernatants were centrifuged at 100,000× *g* for 3 hrs at 4°C. Pellets were either resuspended in culture medium and filtered through 0.45 µm PVDF membrane (Millipore), or loaded on the top of a 20–60% sucrose gradient in phosphate-buffered saline solution (PBS; Quality Biologicals, MD), then centrifuged in a SW55Ti rotor (Beckman) at 100,000× *g* for 16 hrs at 4°C. Gradients were manually harvested from the top in 150 µl fractions. HCVcc (Huh-7.5-produced JFH-1) was obtained by electroporating IVT RNA into Huh-7.5 cells as described [11]. Virus stock was concentrated, aliquoted and stored at −80°C.

### Electron microscopy

BHK-WNV cells (2.5×10^5^) seeded in a 6-well plate were transfected with HCVbp-coding plasmid. Three days later, cells were fixed in 2% glutaraldehyde in 0.1 M sodium cacodylate for 1 hr at RT, then at 4°C, overnight. Cells were subsequently processed for TEM as described [75]. Pooled sucrose fractions containing HCVwt were diluted with PBS then pelleted in Beckman SW55Ti (100,000× *g*, for 2 hr) at 4°C. Pellets were resuspended in 4% paraformaldehyde in PBS and analyzed for negative staining EM. Serum from a cured HCV patient previously infected with genotype 1a was used to detect HCVwt in the immuno-EM analysis.

### Infectivity assay with HCVbp or HCVwt

Virus-containing supernatant from BHK-WNV cells were clarified at 30,000× *g* in SW28 Beckman rotor for 30 min, filtered through 0.45 µm PVDF membranes then concentrated (60-fold) with 10^6^ MWCO Vivaspin filters (Sartorius Stedim, Gottingen, Germany). Huh-7.5 cells (7×10^3^) were seeded in a 8-well chamber coverglass (Lab-Tek II, Nalge Nunc) and incubated with HCVbp for 2 hr at 37°C. After virus inoculum removal, cells were grown for another 48 hr to analyze the expression of HCV NS5A protein. Briefly, cells were washed twice with ice-cold PBS and fixed with 4% paraformaldehyde and 0.15 M sodium cacodylate buffer, pH 7.4, for 20 min at room temperature, followed by washing (5 minutes, twice) with PBS containing 50 mM glycine. After washing with PBS, cells were permeabilized with 0.3% Triton X-100 in PBS for 15 minutes at room temperature, then incubated with blocking solution (10% FBS, 3% BSA, 0.3% Triton X-100 in PBS) for 30 min. Cells were then incubated with primary antibodies: rabbit anti-NS5A IgG and anti-ERGIC-53 mAb (in 1% BSA, 0.1% Triton X-100, in PBS) overnight at 4°C. The fluorescent secondary antibodies were Alexa Fluor 488-conjugated anti-mouse IgG antibody and Alexa Fluor 594- or 635-conjugated anti-rabbit IgG antibodies. Nuclei were labeled with DAPI with antifade (Chemicon, CA). To test the infectivity of HCVwt (1a, 1b and 2a) produced by BHK-WNV cells, HepG2-CD81 cells were seeded on 24-well collagen plates, and the following day, cells were incubated with particles in the presence or absence of IFN-α and ribavirin. Total RNA was harvested daily and intracellular HCV RNA was measured by RT-Taqman PCR.

### Live-cell imaging

Cells were infected with HCV particles containing a genome encoding a tetracysteine-tag (HCVbp-4cys or HCVwt-4cys): Huh-7.5 cells were infected with HCVbp-4cys for 3 days, then incubated with the cell-permeant FIAsH-EDT_2_ or ReAsH-EDT_2_ biarsenical dye according to the manufacturer's protocol (Molecular Probes, Invitrogen). Adding FIAsH (or ReAsH) dye onto live cells expressing TC-tagged proteins should result in a specific fluorescent signal where the tag is present. Samples were observed under a confocal microscope (SP5 X-WLL (white light laser) mono-photon confocal microscope (Leica, Heidelberg, Germany) using a 63× oil immersion objective NA 1.32. Images were deconvolved with Huygens Essential software (Version 5.3, Scientific Volume Imaging BV, Hilversum, The Netherlands). A similar procedure was used to stain cultured human liver slices infected with HCVwt-4cys.

### Bromo-uridine incorporation in Huh-7.5 cells

Huh-7.5 cells (7×10^3^) were seeded in 8-well chamber coverglass and one day later, were infected with HCVbp. At 48 hr post-infection, medium was replaced with D-MEM complete medium containing 2.5 µg/ml actinomycin D (Sigma) for 30 min and transfected with 5-bromo-uridine 5′-triphosphate (BrUTP; Sigma) using Lipofectamine 2000 (Invitrogen). Briefly, 1 µl of Lipofectamine 2000 was added to 10 mM BrUTP, both in 25 µl Opti-MEM I, and incubated for 20 min at room temperature. The BrUTP-Lipofectamine complex was added drop wise onto cells and further incubated for 6 hours. Cells were then fixed, permeabilized and incubated with Alexa Fluor 488 conjugated-anti-BrdU mAb. Confocal microscopy analysis was performed as above.

### RNA analysis and RT-qPCR

Total RNA from sucrose fractions was extracted with Trizol LS (Invitrogen) and RT-TaqMan PCR of HCV 5′-UTR RNA was performed with QuantiTect Probe PCR kit (Qiagen) using IVT RNA standard corresponds to the HCV 5′-UTR. HCV RNA was analyzed directly from infected cells harvested daily using lysis buffer of TaqMan Gene Expression Cells-to-CT kit (Ambion, Applied Biosystems, Invitrogen); RNA was subjected to a RT step followed by HCV TaqMan qPCR analysis performed with HCV specific primers, and HCV 5′-UTR/NH_2_-core *in vitro* transcripts as RT-PCR standards. For HCV and WNV RNA analysis from BHK-WNV cells: Total RNA was extracted from cells and pelleted supernatants with Trizol LS followed by RT using random hexamer and Superscript III at 50°C, for 1 hr. *Renilla* luciferase-specific primers as the target gene for WNV-SG rep RNA. See Text S1 for details.

### Determination of HCVbp (or HCVcc) TCID_50_

The released particles were filtered, concentrated and serially diluted before incubated with Huh-7.5 cells for 3–4 days. NS5A-positive cells were analyzed by immunofluorescence and the number of positive cells was determined using *Odyssey* In-cell Western system (Li-Cor Biosciences, Lincoln, NE). See Text S1 for details.

### Transduction of HepG2 cells with hCD81-lentivirus

The cDNA of human CD81 (hCD81) from Huh-7.5 cells were amplified by reverse transcription (RT)-PCR and cloned into pENTR 2B (Invitrogen) followed by recombination with pLenti6.2/V5-DEST (Invitrogen) to according to manufacturer's recommendation. See Text S1 for details.

### Infection of cultured human liver slices

Human liver slices were infected with HCVcc (JFH-1) produced in Huh-7.5 cells, HCVwt produced in BHK-WNV cells, or not infected. Six-to eight days after infection, co-immunostaining was performed with HCV serum or monoclonal antibodies, followed by DyLight 488 conjugated-anti-human IgG F(ab′)2 (Jackson ImmunoResearch Laboratories, West Grove, PA), or Alexa Fluor 546 conjugated-anti-mouse IgG goat antibody (Invitrogen). Liver slices were analyzed with a mono-photon multi-focal confocal microscope (Leica SP5 Resonant Scanner, Heidelberg, Germany) coupled to a high resolution CCD. For live-cell staining, human liver slices were infected with HCVwt-4cys (HCVwt encoding a tetracysteine tag) for 6 days, incubated with the cell-permeant TC-FIAsH dye and analyzed (over a thickness of 100–150 µm) as above, using a multi-photon mono-focal confocal microscope (Leica TCS SP5 Resonant Scanner, Heidelberg, Germany).

### HCVrp entry assay with the reporter system and production of WNV reporter particles

See Text S1 for details.

### Huh-7.5 cell gene knockdown using siRNAs

See Text S1 for details.

### Ethics statement

All human samples were obtained during routine medical care and in compliance with the standard Ethical Guidelines of the Institutional Review Board of Cochin Hospital (Paris) that approved the study.

## Supporting Information

Figure S1 Inhibition of HCV structural proteins release by HCV and SFV subgenomic replicons in Huh-7.5 and BHK-21 cells, respectively.
(0.69 MB PPT)

Figure S2 Immuno-EM of BHK-WNV cells transfected with HCVbp-coding plasmid.
(3.70 MB PPT)

Figure S3 A. Detection of HCV core, E1, E2 of HCVbp after fractionation on a 20–60% sucrose gradient. B. Effect of brefeldin A on HCV release. C. HCV release required maturation of viral glycoproteins. D. WNV, but not HCV, structural proteins *trans*-encapsidate WNV SG-replicon that is infectious in Huh-7.5 cells. E. Effect of antiviral treatment of WNV SG-replicon on HCV release by BHK-WNV cells.
(4.56 MB PPT)

Figure S4 A. Co-localization of 4cys-HCVbp with lipid droplets in BHK-WNV cells. B. Pre-incubation of HCVbp with serum from HCV-cured patient abolished its infectivity. C. Specificity of in-house HCV NS5A rabbit polyclonal antibody. D. Infection of Huh-7.5 cells with HCVbp-4cys.
(1.12 MB PPT)

Figure S5 A. Surface expression of human CD81 by HepG2-CD81 cells. B. BHK-WNV cells, but not parental BHK-21, produced infectious HCVrp. C. HCV receptors knockdown by siRNA in Huh-7.5 cells. D. Inhibition of HCVrp entry by anti-CD81 and anti-SR-BI antibodies. E. Inhibition of HCVrp entry by anti-HVR-1 antibodies. F. Effect of NS2 on the buoyant densities and infectivity of HCVrp.
(0.70 MB PPT)

Figure S6 Control liver slices with secondary antibodies.
(3.18 MB PPT)

Table S1 Correlation between HCVrp infectivity and candidate receptor expression in several cell lines.
(0.17 MB PPT)

Text S1 Supporting Data; Supporting Materials and Methods; Supporting References.
(0.07 MB DOC)
