# Supplementary material for: A New Model to Produce Infectious Hepatitis C Virus without the Replication Requirement
Source: PLoS Pathog. 2011 Apr 14;7(4):e1001333. doi: 10.1371/journal.ppat.1001333 (PMC3077361; doi:10.1371/journal.ppat.1001333)
Supplement: Figure S1 — Inhibition of HCV structural proteins release by HCV and SFV subgenomic replicons in Huh-7.5 and BHK-21 cells, respectively. (0.69 MB PPT) [file ppat.1001333.s001.ppt]

## Slide 1
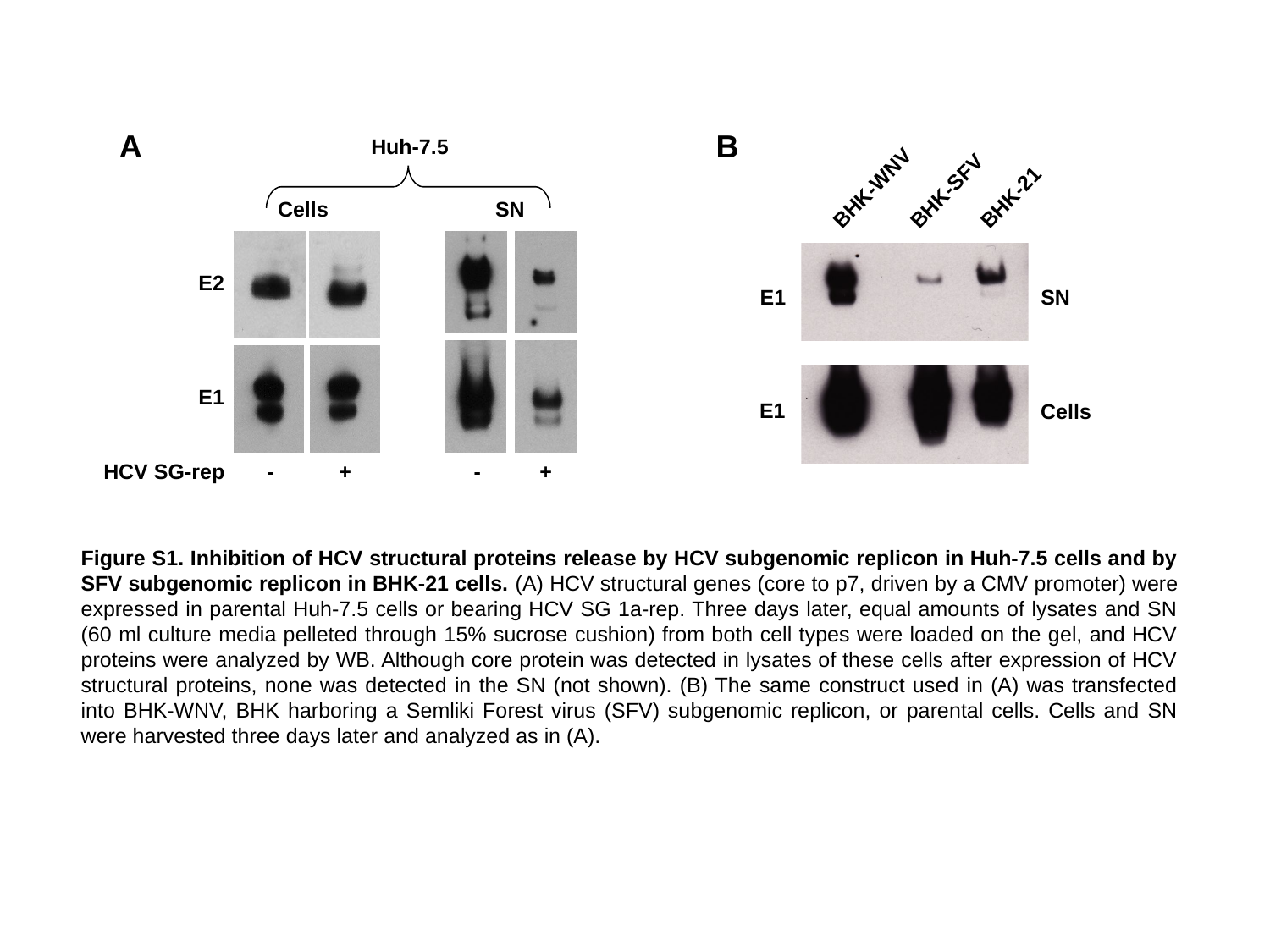

A
Huh-7.5
 Cells
SN
E2
E1
HCV SG-rep
- +
- +
B
BHK-WNV
BHK-SFV
BHK-21
E1
SN
E1
Cells
Figure S1. Inhibition of HCV structural proteins release by HCV subgenomic replicon in Huh-7.5 cells and by SFV subgenomic replicon in BHK-21 cells. (A) HCV structural genes (core to p7, driven by a CMV promoter) were expressed in parental Huh-7.5 cells or bearing HCV SG 1a-rep. Three days later, equal amounts of lysates and SN (60 ml culture media pelleted through 15% sucrose cushion) from both cell types were loaded on the gel, and HCV proteins were analyzed by WB. Although core protein was detected in lysates of these cells after expression of HCV structural proteins, none was detected in the SN (not shown). (B) The same construct used in (A) was transfected into BHK-WNV, BHK harboring a Semliki Forest virus (SFV) subgenomic replicon, or parental cells. Cells and SN were harvested three days later and analyzed as in (A).
