# Supplementary material for: A New Model to Produce Infectious Hepatitis C Virus without the Replication Requirement
Source: PLoS Pathog. 2011 Apr 14;7(4):e1001333. doi: 10.1371/journal.ppat.1001333 (PMC3077361; doi:10.1371/journal.ppat.1001333)
Supplement: Figure S2 — Immuno-EM of BHK-WNV cells transfected with HCVbp-coding plasmid. (3.70 MB PPT) [file ppat.1001333.s002.ppt]

## Slide 1
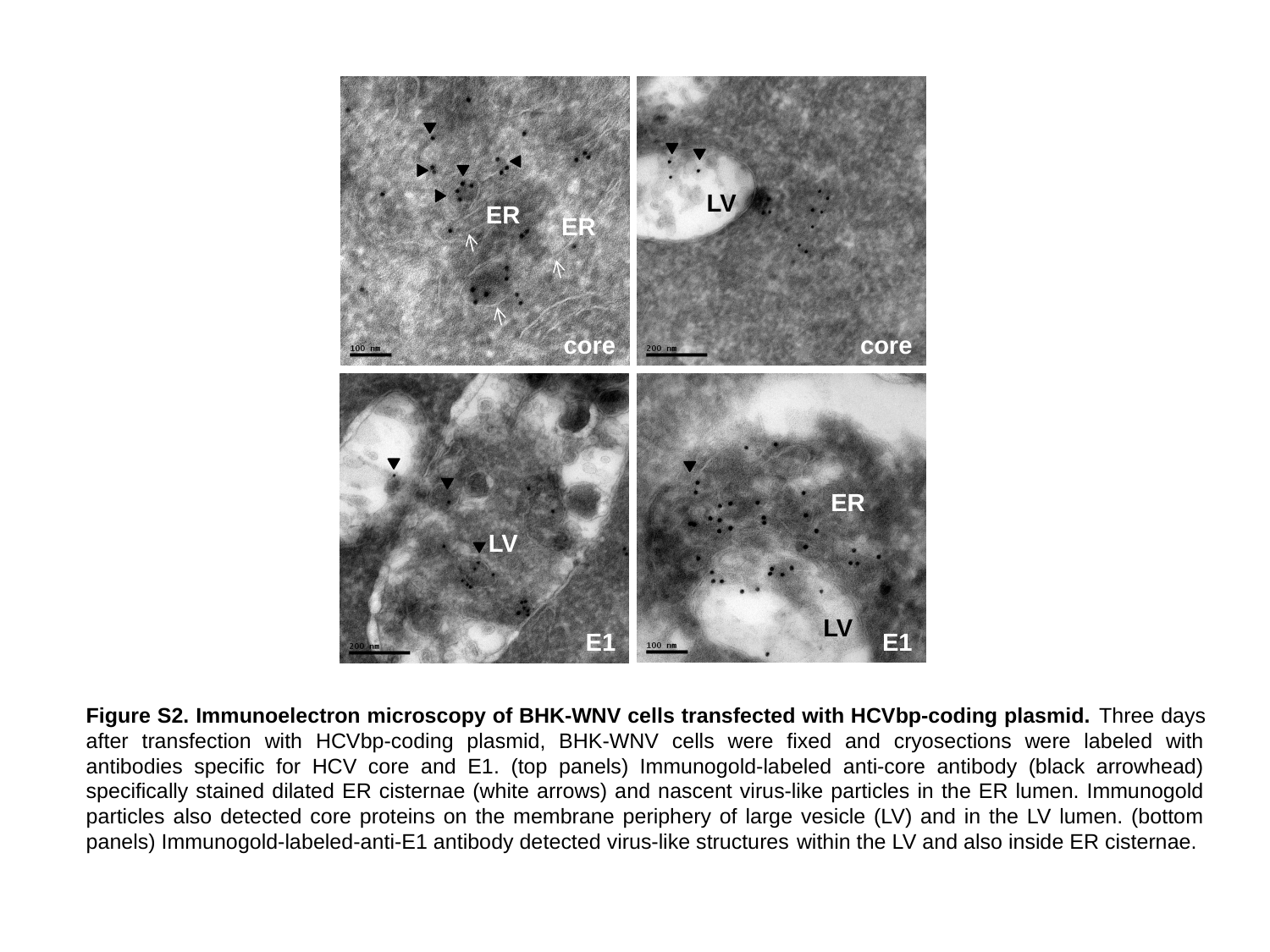

LV
ER
ER
core
core
LV
ER
LV
LV
E1
E1
E1
Figure S2. Immunoelectron microscopy of BHK-WNV cells transfected with HCVbp-coding plasmid. Three days after transfection with HCVbp-coding plasmid, BHK-WNV cells were fixed and cryosections were labeled with antibodies specific for HCV core and E1. (top panels) Immunogold-labeled anti-core antibody (black arrowhead) specifically stained dilated ER cisternae (white arrows) and nascent virus-like particles in the ER lumen. Immunogold particles also detected core proteins on the membrane periphery of large vesicle (LV) and in the LV lumen. (bottom panels) Immunogold-labeled-anti-E1 antibody detected virus-like structures within the LV and also inside ER cisternae.
