# Supplementary material for: A New Model to Produce Infectious Hepatitis C Virus without the Replication Requirement
Source: PLoS Pathog. 2011 Apr 14;7(4):e1001333. doi: 10.1371/journal.ppat.1001333 (PMC3077361; doi:10.1371/journal.ppat.1001333)
Supplement: Figure S3 — A. Detection of HCV core, E1, E2 of HCVbp after fractionation on a 20–60% sucrose gradient. B. Effect of brefeldin A on HCV release. C. HCV release required maturation of viral glycoproteins. D. WNV, but not HCV, structural proteins trans-encapsidate WNV SG-replicon that is infectious in Huh-7.5 cells. E. Effect of antiviral treatment of WNV SG-replicon on HCV release by BHK-WNV cells. (4.56 MB PPT) [file ppat.1001333.s003.ppt]

## Slide 1
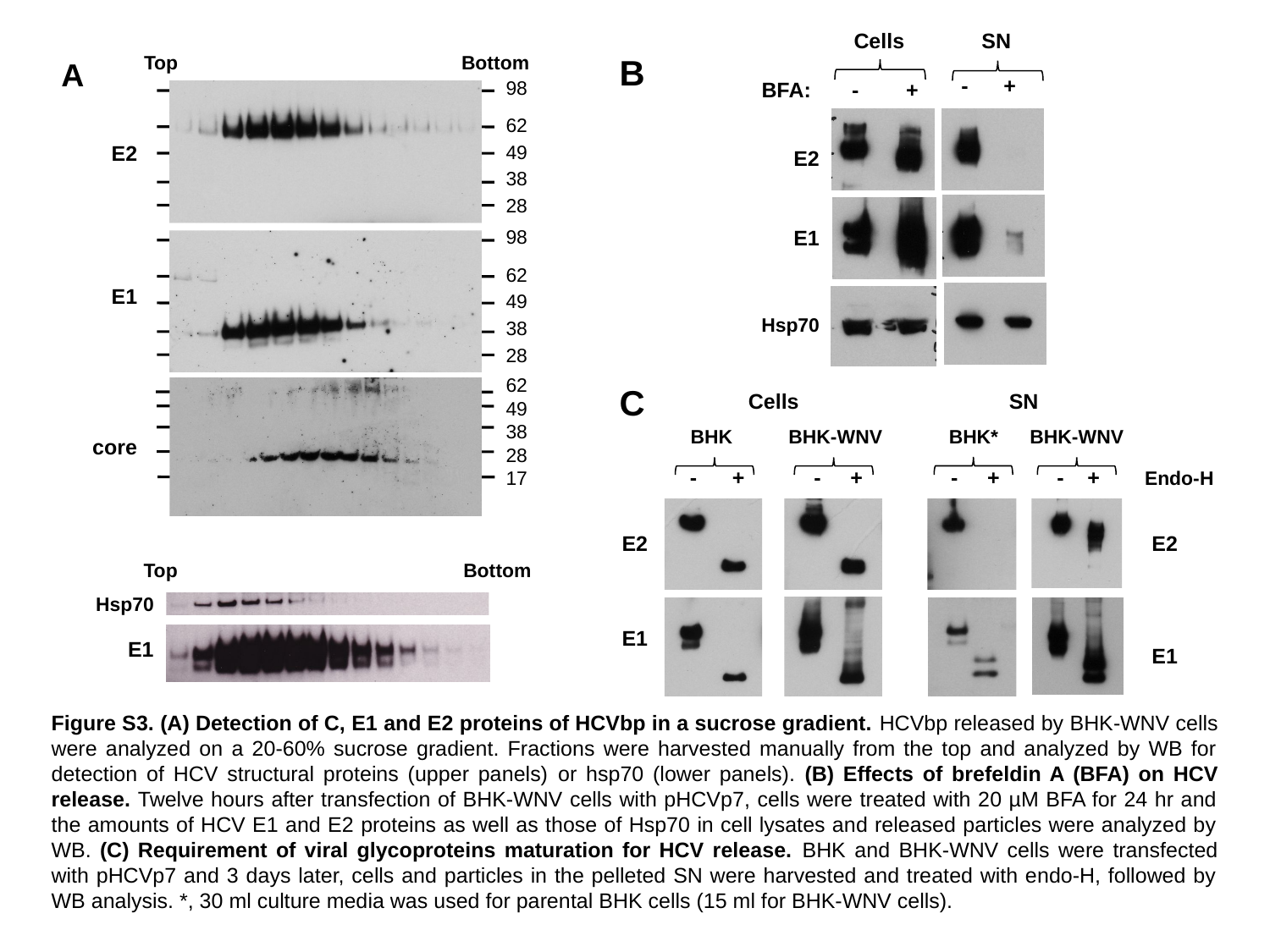

Cells
SN
 - +
BFA:
 - +
E2
E1
Hsp70
Top					Bottom
98
62
49
38
28
98
62
49
38
28
62
49
38
28
17
E2
E1
core
B
A
C
Cells
SN
BHK
BHK-WNV
BHK*
BHK-WNV
 - +
- +
 - +
- +
Endo-H
E2
E2
E1
E1
Top				 Bottom
Hsp70
E1
Figure S3. (A) Detection of C, E1 and E2 proteins of HCVbp in a sucrose gradient. HCVbp released by BHK-WNV cells were analyzed on a 20-60% sucrose gradient. Fractions were harvested manually from the top and analyzed by WB for detection of HCV structural proteins (upper panels) or hsp70 (lower panels). (B) Effects of brefeldin A (BFA) on HCV release. Twelve hours after transfection of BHK-WNV cells with pHCVp7, cells were treated with 20 µM BFA for 24 hr and the amounts of HCV E1 and E2 proteins as well as those of Hsp70 in cell lysates and released particles were analyzed by WB. (C) Requirement of viral glycoproteins maturation for HCV release. BHK and BHK-WNV cells were transfected with pHCVp7 and 3 days later, cells and particles in the pelleted SN were harvested and treated with endo-H, followed by WB analysis. *, 30 ml culture media was used for parental BHK cells (15 ml for BHK-WNV cells).

## Slide 2
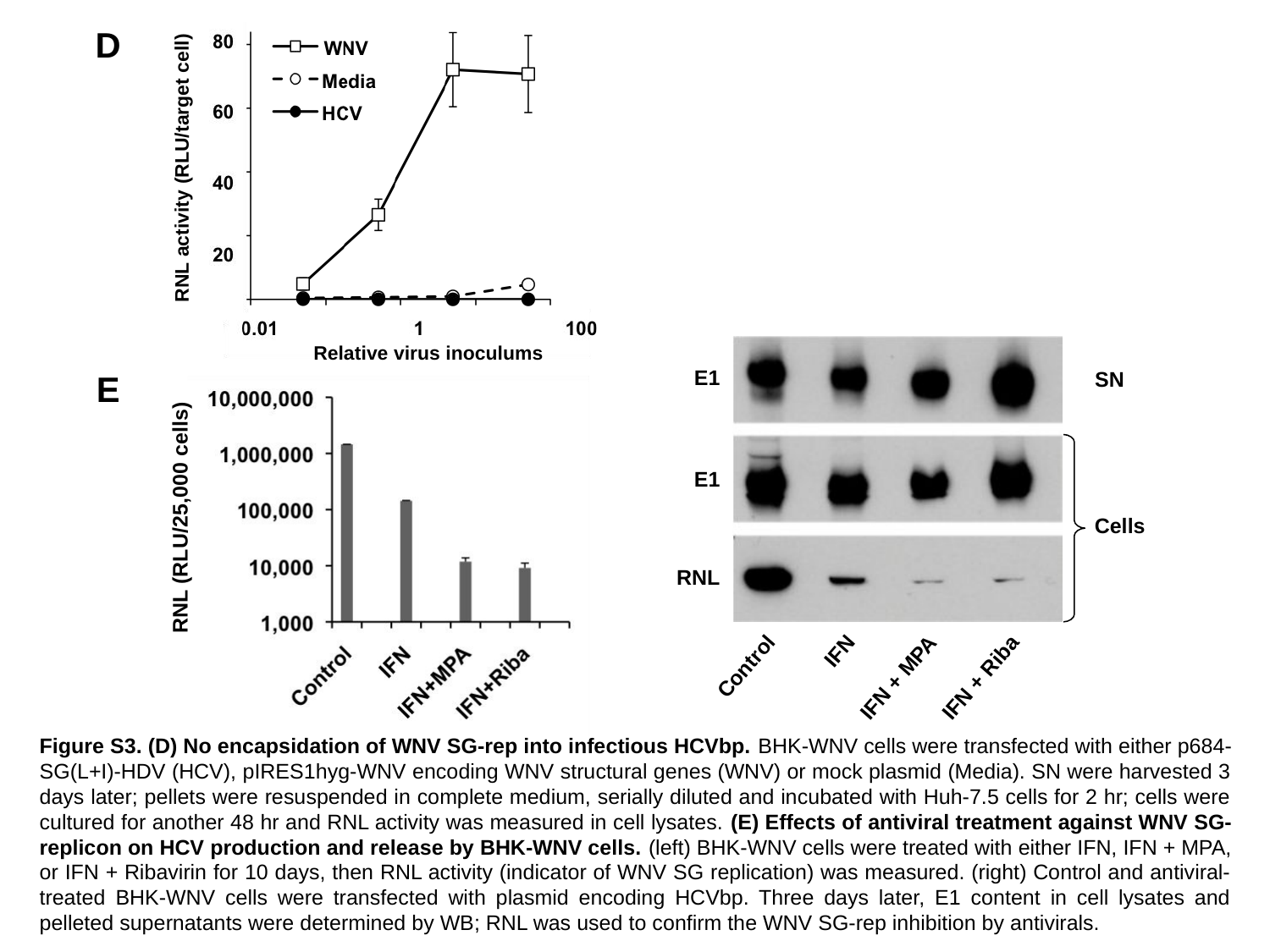

D
RNL activity (RLU/target cell)
Relative virus inoculums
E1
E1
RNL
SN
E
RNL (RLU/25,000 cells)
Cells
IFN
Control
Figure S3. (D) No encapsidation of WNV SG-rep into infectious HCVbp. BHK-WNV cells were transfected with either p684-SG(L+I)-HDV (HCV), pIRES1hyg-WNV encoding WNV structural genes (WNV) or mock plasmid (Media). SN were harvested 3 days later; pellets were resuspended in complete medium, serially diluted and incubated with Huh-7.5 cells for 2 hr; cells were cultured for another 48 hr and RNL activity was measured in cell lysates. (E) Effects of antiviral treatment against WNV SG-replicon on HCV production and release by BHK-WNV cells. (left) BHK-WNV cells were treated with either IFN, IFN + MPA, or IFN + Ribavirin for 10 days, then RNL activity (indicator of WNV SG replication) was measured. (right) Control and antiviral-treated BHK-WNV cells were transfected with plasmid encoding HCVbp. Three days later, E1 content in cell lysates and pelleted supernatants were determined by WB; RNL was used to confirm the WNV SG-rep inhibition by antivirals.
IFN + Riba
IFN + MPA
