# Supplementary material for: A New Model to Produce Infectious Hepatitis C Virus without the Replication Requirement
Source: PLoS Pathog. 2011 Apr 14;7(4):e1001333. doi: 10.1371/journal.ppat.1001333 (PMC3077361; doi:10.1371/journal.ppat.1001333)
Supplement: Figure S4 — A. Co-localization of 4cys-HCVbp with lipid droplets in BHK-WNV cells. B. Pre-incubation of HCVbp with serum from HCV-cured patient abolished its infectivity. C. Specificity of in-house HCV NS5A rabbit polyclonal antibody. D. Infection of Huh-7.5 cells with HCVbp-4cys. (1.12 MB PPT) [file ppat.1001333.s004.ppt]

## Slide 1
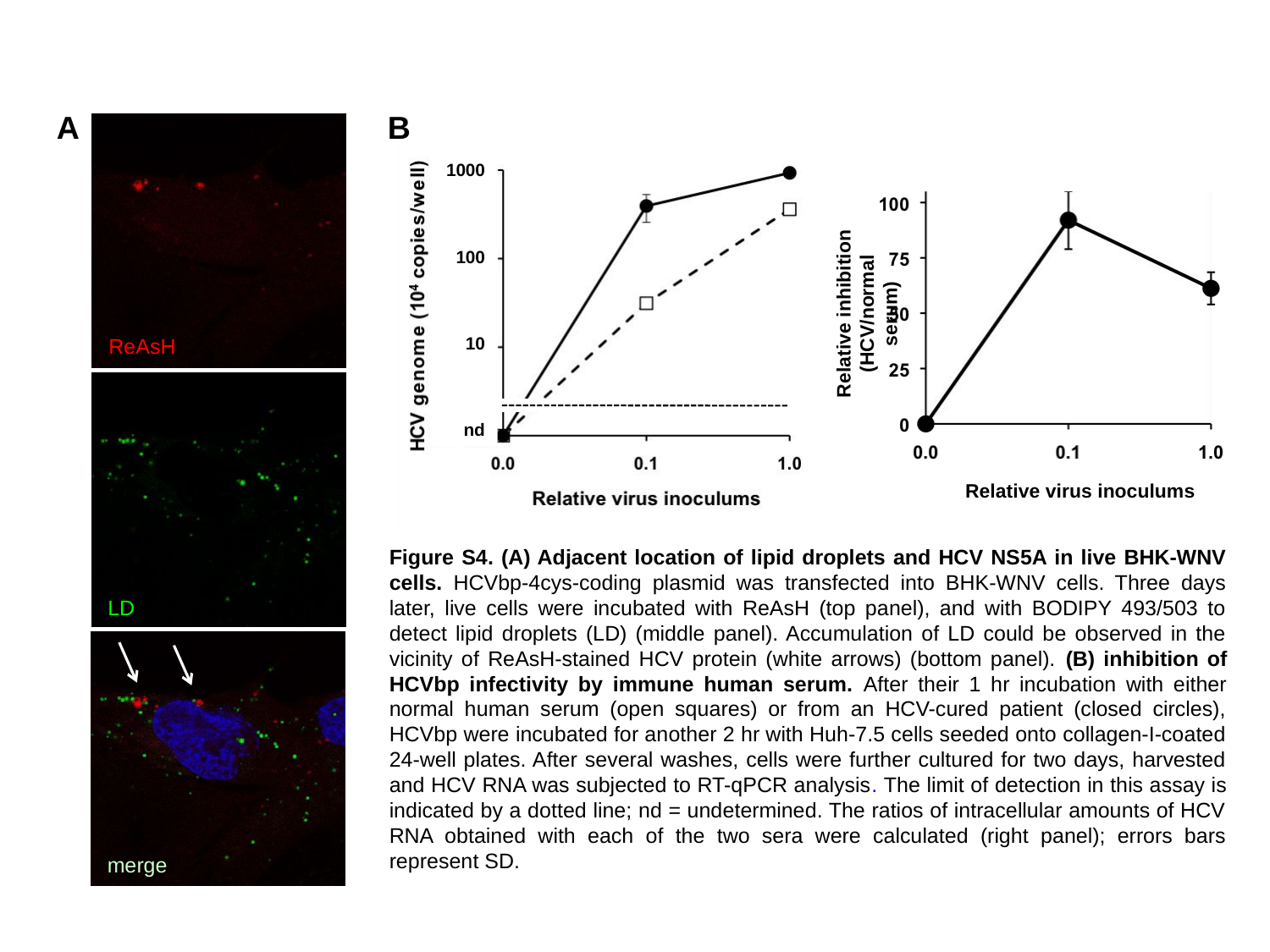

A
B
ReAsH
LD
merge
1000
100
10
nd
Relative inhibition (HCV/normal serum)
Relative virus inoculums
Figure S4. (A) Adjacent location of lipid droplets and HCV NS5A in live BHK-WNV cells. HCVbp-4cys-coding plasmid was transfected into BHK-WNV cells. Three days later, live cells were incubated with ReAsH (top panel), and with BODIPY 493/503 to detect lipid droplets (LD) (middle panel). Accumulation of LD could be observed in the vicinity of ReAsH-stained HCV protein (white arrows) (bottom panel). (B) inhibition of HCVbp infectivity by immune human serum. After their 1 hr incubation with either normal human serum (open squares) or from an HCV-cured patient (closed circles), HCVbp were incubated for another 2 hr with Huh-7.5 cells seeded onto collagen-I-coated 24-well plates. After several washes, cells were further cultured for two days, harvested and HCV RNA was subjected to RT-qPCR analysis. The limit of detection in this assay is indicated by a dotted line; nd = undetermined. The ratios of intracellular amounts of HCV RNA obtained with each of the two sera were calculated (right panel); errors bars represent SD.

## Slide 2
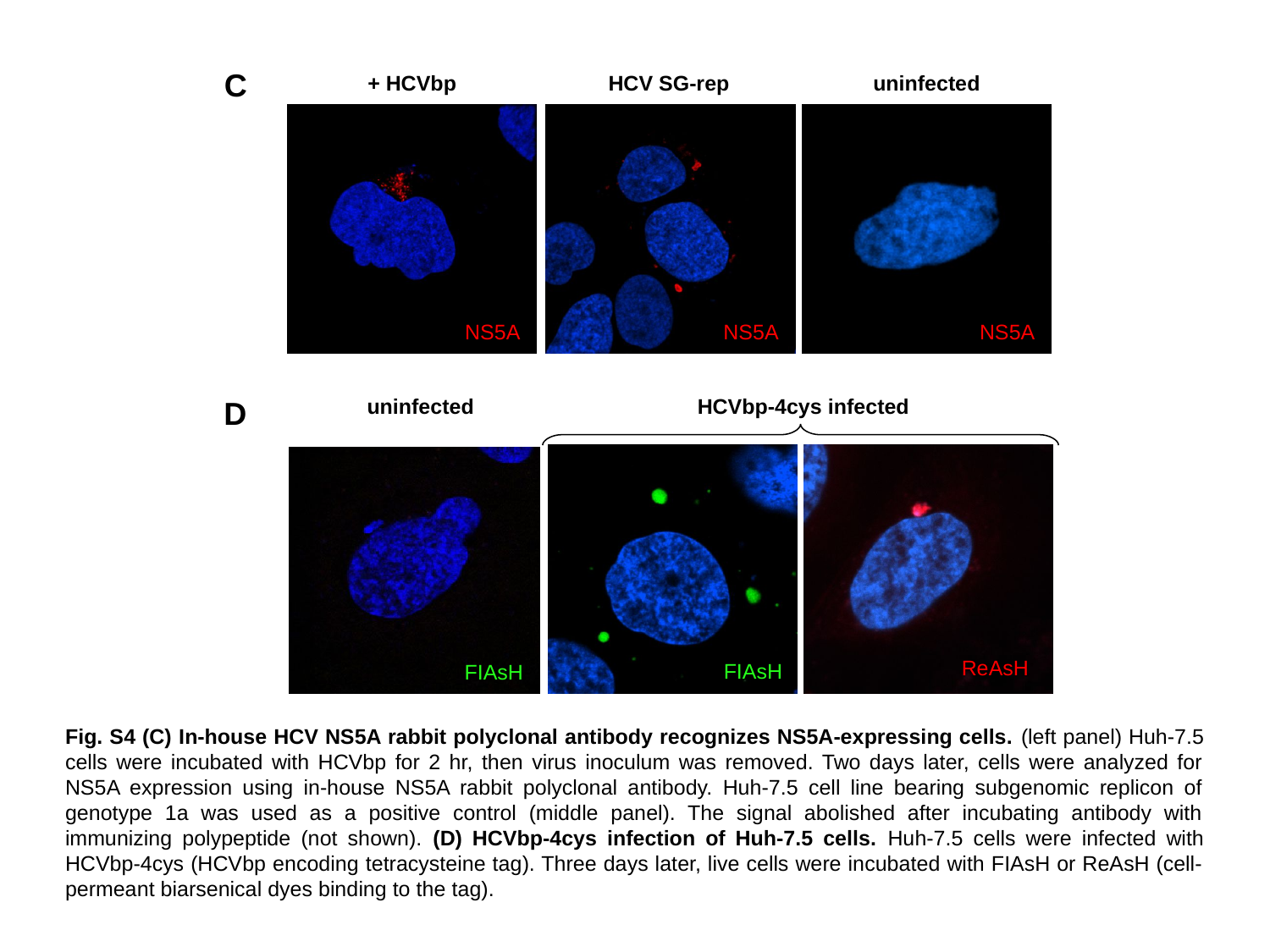

C
+ HCVbp
HCV SG-rep
uninfected
NS5A
NS5A
NS5A
uninfected
HCVbp-4cys infected
ReAsH
FIAsH
FIAsH
D
Fig. S4 (C) In-house HCV NS5A rabbit polyclonal antibody recognizes NS5A-expressing cells. (left panel) Huh-7.5 cells were incubated with HCVbp for 2 hr, then virus inoculum was removed. Two days later, cells were analyzed for NS5A expression using in-house NS5A rabbit polyclonal antibody. Huh-7.5 cell line bearing subgenomic replicon of genotype 1a was used as a positive control (middle panel). The signal abolished after incubating antibody with immunizing polypeptide (not shown). (D) HCVbp-4cys infection of Huh-7.5 cells. Huh-7.5 cells were infected with HCVbp-4cys (HCVbp encoding tetracysteine tag). Three days later, live cells were incubated with FIAsH or ReAsH (cell-permeant biarsenical dyes binding to the tag).
