# Supplementary material for: A New Model to Produce Infectious Hepatitis C Virus without the Replication Requirement
Source: PLoS Pathog. 2011 Apr 14;7(4):e1001333. doi: 10.1371/journal.ppat.1001333 (PMC3077361; doi:10.1371/journal.ppat.1001333)
Supplement: Figure S5 — A. Surface expression of human CD81 by HepG2-CD81 cells. B. BHK-WNV cells, but not parental BHK-21, produced infectious HCVrp. C. HCV receptors knockdown by siRNA in Huh-7.5 cells. D. Inhibition of HCVrp entry by anti-CD81 and anti-SR-BI antibodies. E. Inhibition of HCVrp entry by anti-HVR-1 antibodies. F. Effect of NS2 on the buoyant densities and infectivity of HCVrp. (0.70 MB PPT) [file ppat.1001333.s005.ppt]

## Slide 1
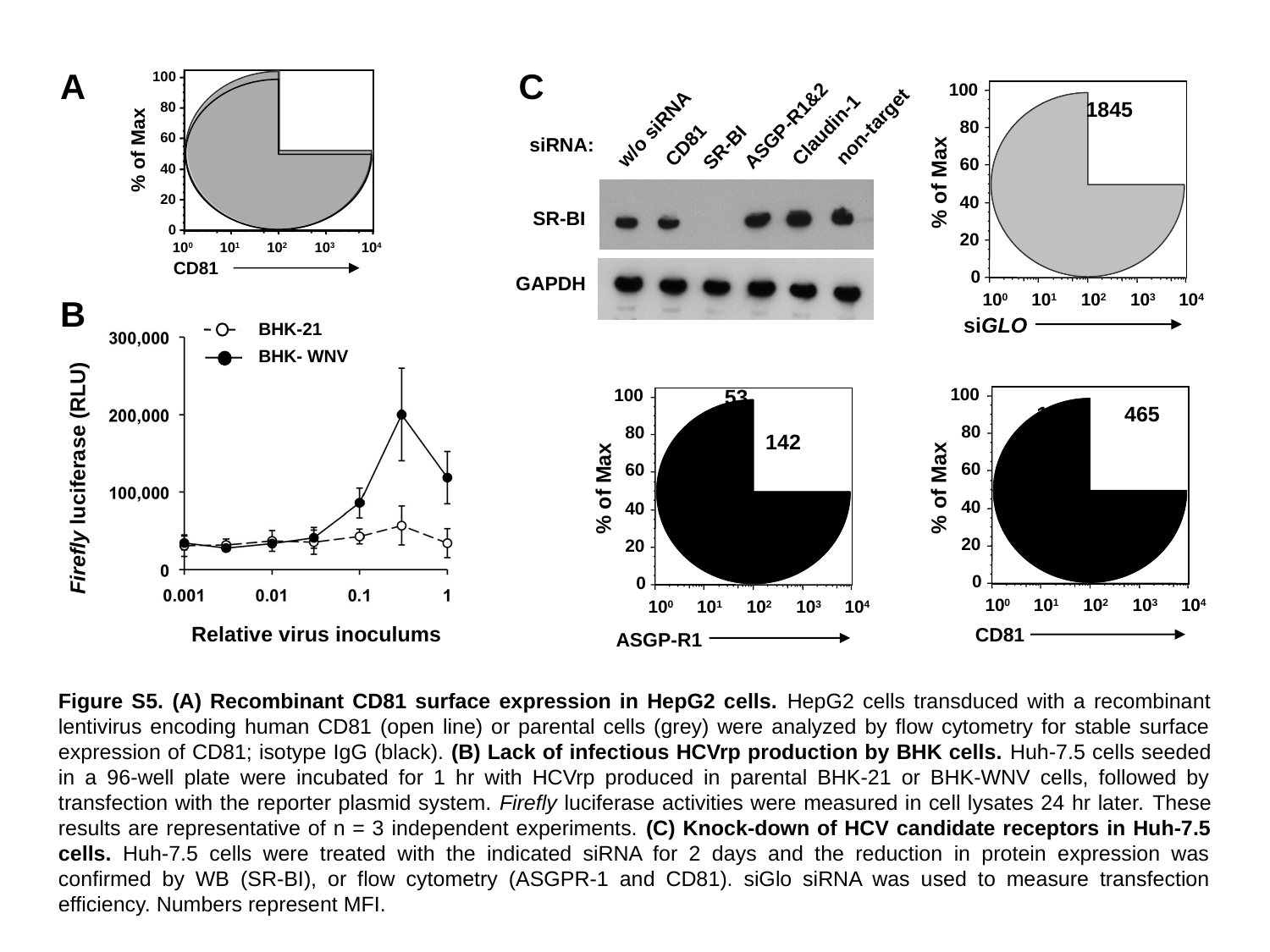

A
C
ASGP-R1&2
non-target
w/o siRNA
Claudin-1
CD81
siRNA:
SR-BI
SR-BI
GAPDH
100
80
60
40
20
0
100
101
102
103
104
CD81
100
80
60
40
20
0
1845
% of Max
100
101
102
103
104
siGLO
% of Max
B
BHK-21
BHK- WNV
Firefly luciferase (RLU)
Relative virus inoculums
53
100
80
60
40
20
0
142
% of Max
100
101
102
103
104
100
80
60
40
20
0
123
465
% of Max
100
101
102
103
104
CD81
ASGP-R1
Figure S5. (A) Recombinant CD81 surface expression in HepG2 cells. HepG2 cells transduced with a recombinant lentivirus encoding human CD81 (open line) or parental cells (grey) were analyzed by flow cytometry for stable surface expression of CD81; isotype IgG (black). (B) Lack of infectious HCVrp production by BHK cells. Huh-7.5 cells seeded in a 96-well plate were incubated for 1 hr with HCVrp produced in parental BHK-21 or BHK-WNV cells, followed by transfection with the reporter plasmid system. Firefly luciferase activities were measured in cell lysates 24 hr later. These results are representative of n = 3 independent experiments. (C) Knock-down of HCV candidate receptors in Huh-7.5 cells. Huh-7.5 cells were treated with the indicated siRNA for 2 days and the reduction in protein expression was confirmed by WB (SR-BI), or flow cytometry (ASGPR-1 and CD81). siGlo siRNA was used to measure transfection efficiency. Numbers represent MFI.

## Slide 2
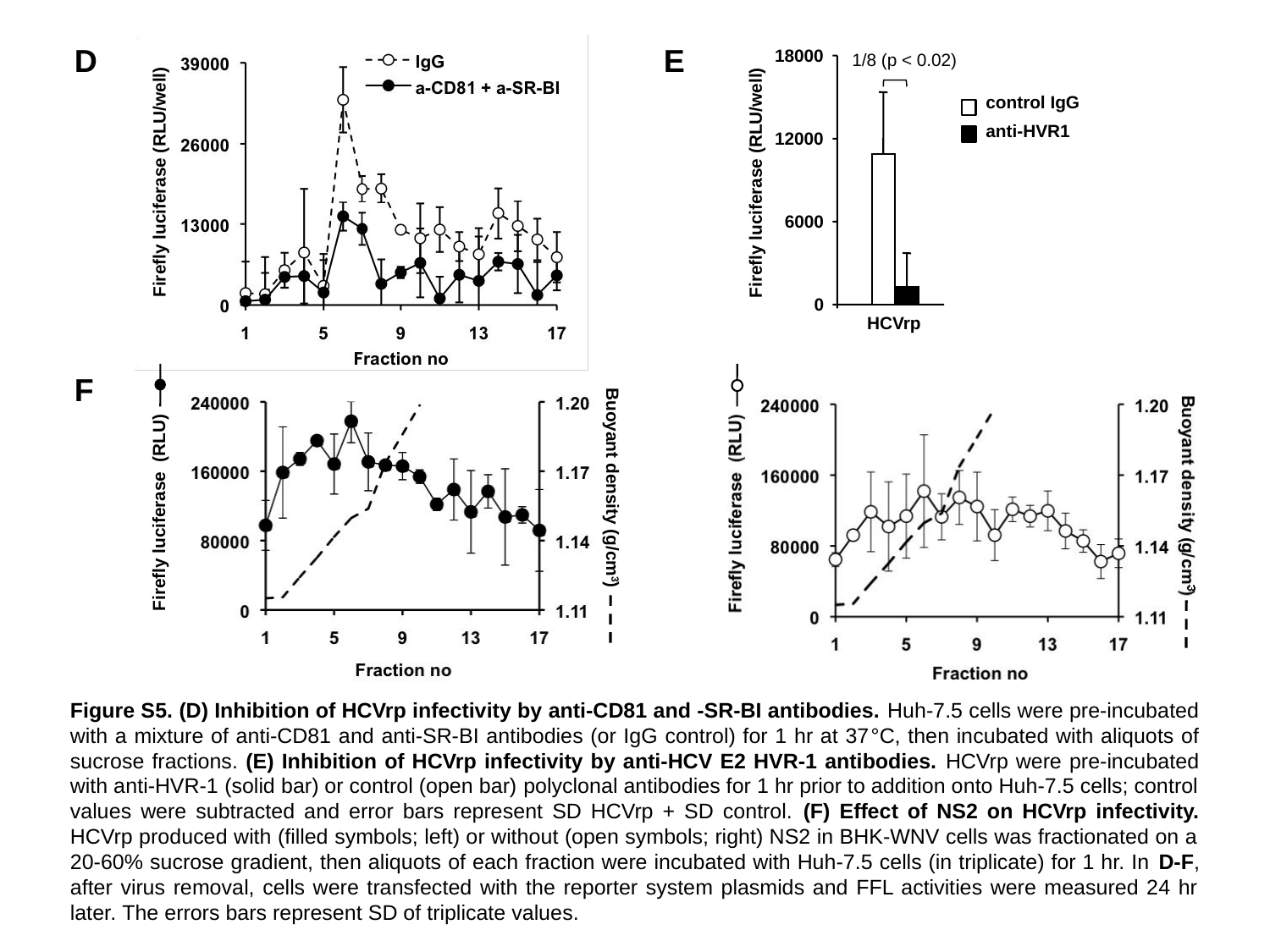

D
Firefly luciferase (RLU/well)
E
1/8 (p < 0.02)
18000
control IgG
anti-HVR1
12000
Firefly luciferase (RLU/well)
6000
0
HCVrp
F
Buoyant density (g/cm3)
Firefly luciferase (RLU)
Figure S5. (D) Inhibition of HCVrp infectivity by anti-CD81 and -SR-BI antibodies. Huh-7.5 cells were pre-incubated with a mixture of anti-CD81 and anti-SR-BI antibodies (or IgG control) for 1 hr at 37°C, then incubated with aliquots of sucrose fractions. (E) Inhibition of HCVrp infectivity by anti-HCV E2 HVR-1 antibodies. HCVrp were pre-incubated with anti-HVR-1 (solid bar) or control (open bar) polyclonal antibodies for 1 hr prior to addition onto Huh-7.5 cells; control values were subtracted and error bars represent SD HCVrp + SD control. (F) Effect of NS2 on HCVrp infectivity. HCVrp produced with (filled symbols; left) or without (open symbols; right) NS2 in BHK-WNV cells was fractionated on a 20-60% sucrose gradient, then aliquots of each fraction were incubated with Huh-7.5 cells (in triplicate) for 1 hr. In D-F, after virus removal, cells were transfected with the reporter system plasmids and FFL activities were measured 24 hr later. The errors bars represent SD of triplicate values.
