# Supplementary material for: A New Model to Produce Infectious Hepatitis C Virus without the Replication Requirement
Source: PLoS Pathog. 2011 Apr 14;7(4):e1001333. doi: 10.1371/journal.ppat.1001333 (PMC3077361; doi:10.1371/journal.ppat.1001333)
Supplement: Figure S6 — Control liver slices with secondary antibodies. (3.18 MB PPT) [file ppat.1001333.s006.ppt]

## Slide 1
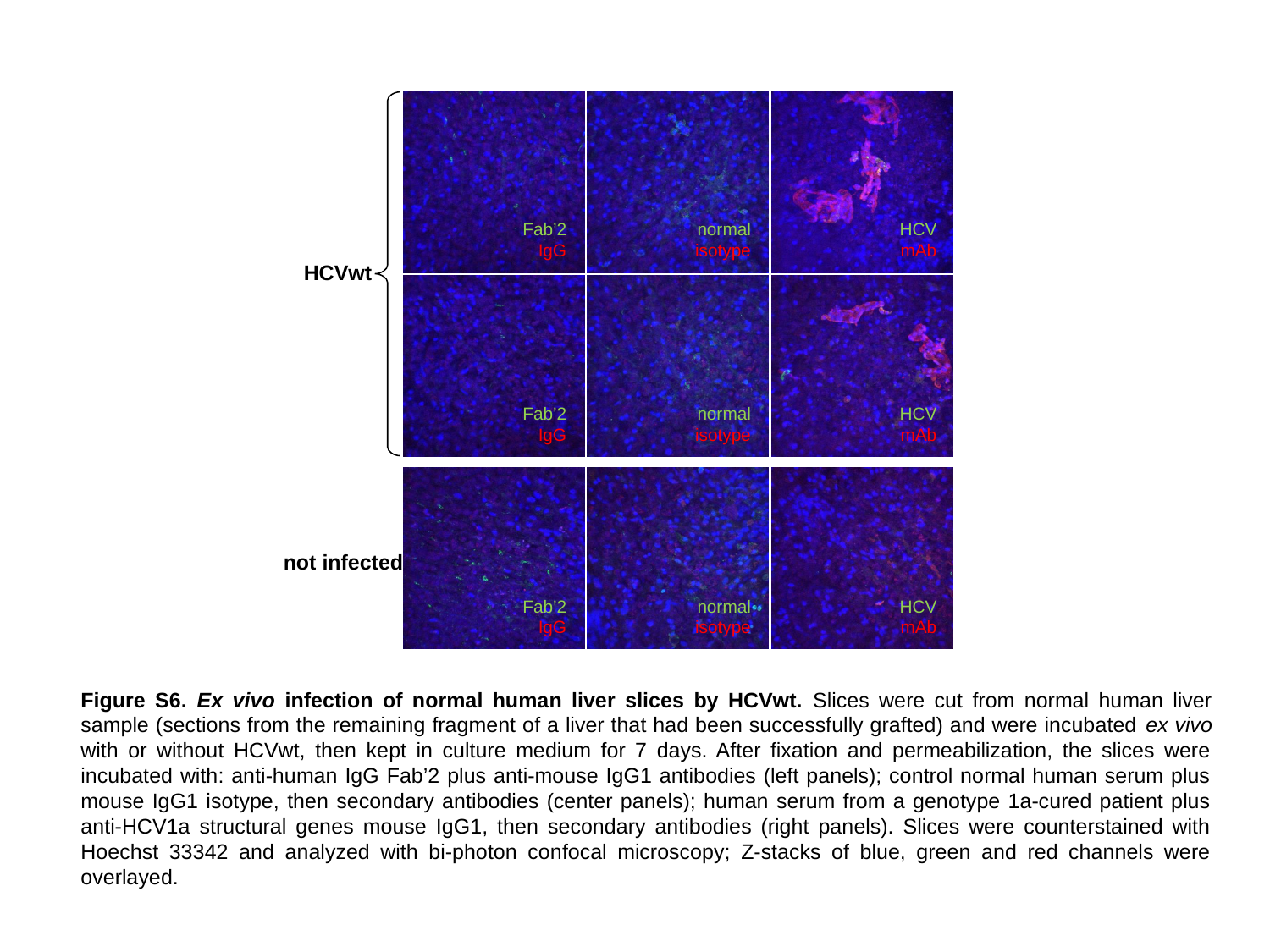

Fab’2
IgG
normal
isotype
HCV
mAb
HCVwt
Fab’2
IgG
normal
isotype
HCV
mAb
not infected
Fab’2
IgG
normal
isotype
HCV
mAb
Figure S6. Ex vivo infection of normal human liver slices by HCVwt. Slices were cut from normal human liver sample (sections from the remaining fragment of a liver that had been successfully grafted) and were incubated ex vivo with or without HCVwt, then kept in culture medium for 7 days. After fixation and permeabilization, the slices were incubated with: anti-human IgG Fab’2 plus anti-mouse IgG1 antibodies (left panels); control normal human serum plus mouse IgG1 isotype, then secondary antibodies (center panels); human serum from a genotype 1a-cured patient plus anti-HCV1a structural genes mouse IgG1, then secondary antibodies (right panels). Slices were counterstained with Hoechst 33342 and analyzed with bi-photon confocal microscopy; Z-stacks of blue, green and red channels were overlayed.
