# Supplementary material for: A New Model to Produce Infectious Hepatitis C Virus without the Replication Requirement
Source: PLoS Pathog. 2011 Apr 14;7(4):e1001333. doi: 10.1371/journal.ppat.1001333 (PMC3077361; doi:10.1371/journal.ppat.1001333)
Supplement: Table S1 — Correlation between HCVrp infectivity and candidate receptor expression in several cell lines. (0.17 MB PPT) [file ppat.1001333.s007.ppt]

## Slide 1
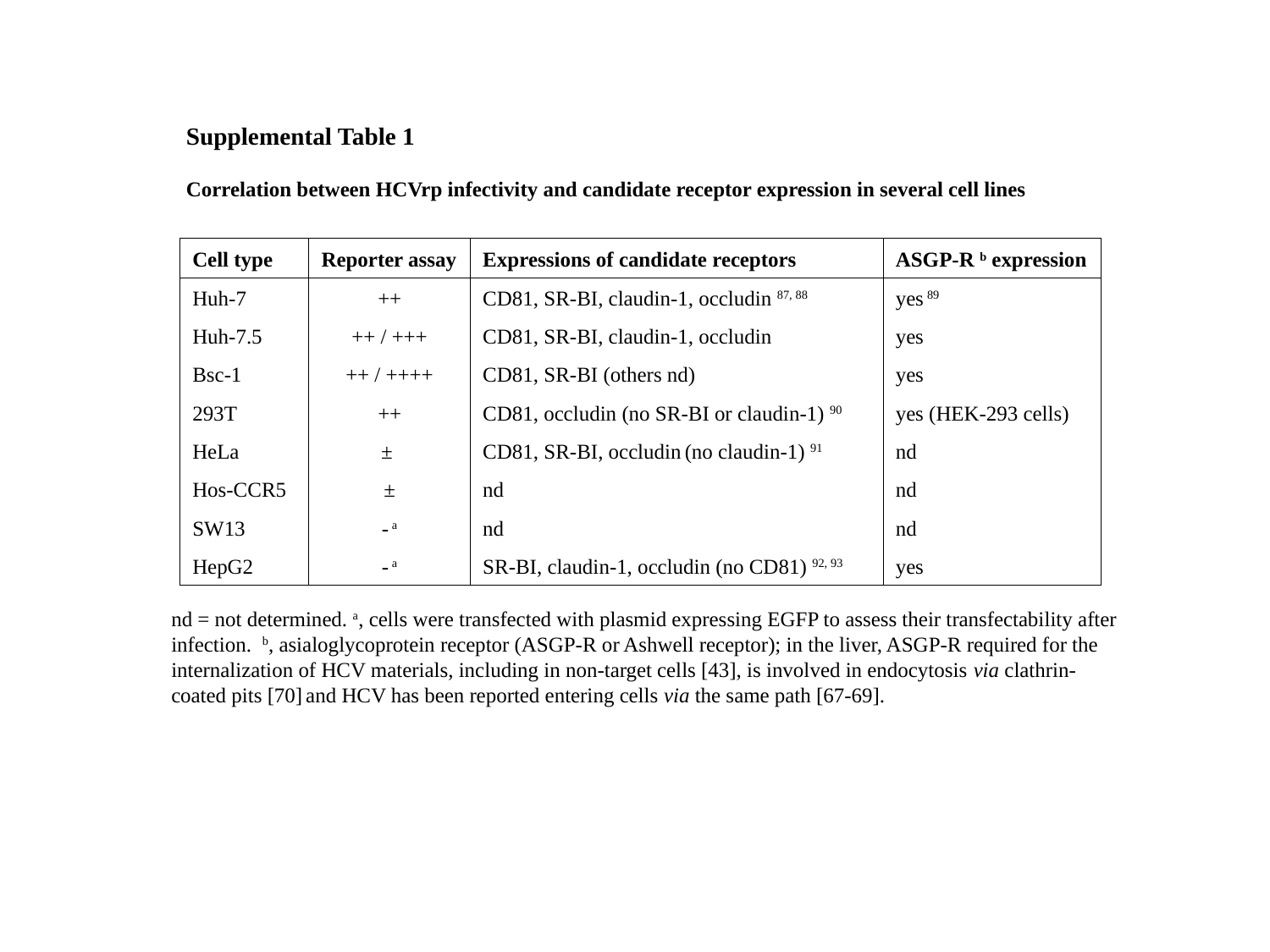

Supplemental Table 1
Correlation between HCVrp infectivity and candidate receptor expression in several cell lines
| Cell type | Reporter assay | Expressions of candidate receptors | ASGP-R b expression |
| --- | --- | --- | --- |
| Huh-7 | ++ | CD81, SR-BI, claudin-1, occludin 87, 88 | yes 89 |
| Huh-7.5 | ++ / +++ | CD81, SR-BI, claudin-1, occludin | yes |
| Bsc-1 | ++ / ++++ | CD81, SR-BI (others nd) | yes |
| 293T | ++ | CD81, occludin (no SR-BI or claudin-1) 90 | yes (HEK-293 cells) |
| HeLa | ± | CD81, SR-BI, occludin (no claudin-1) 91 | nd |
| Hos-CCR5 | ± | nd | nd |
| SW13 | - a | nd | nd |
| HepG2 | - a | SR-BI, claudin-1, occludin (no CD81) 92, 93 | yes |
nd = not determined. a, cells were transfected with plasmid expressing EGFP to assess their transfectability after infection. b, asialoglycoprotein receptor (ASGP-R or Ashwell receptor); in the liver, ASGP-R required for the internalization of HCV materials, including in non-target cells [43], is involved in endocytosis via clathrin-coated pits [70] and HCV has been reported entering cells via the same path [67-69].
