# Supplementary material for: A New Model to Produce Infectious Hepatitis C Virus without the Replication Requirement
Source: PLoS Pathog. 2011 Apr 14;7(4):e1001333. doi: 10.1371/journal.ppat.1001333 (PMC3077361; doi:10.1371/journal.ppat.1001333)
Supplement: Text S1 — Supporting Data; Supporting Materials and Methods; Supporting References. (0.07 MB DOC) [file ppat.1001333.s008.doc]

**INVENTORY OF SUPPORTING INFORMATION**

**Supporting Figures**

**Figure S**1. Inhibition of HCV structural proteins release by HCV and SFV subgenomic replicons in Huh-7.5 and BHK-21 cells, respectively.

**Figure S2**. Immuno-EM of BHK-WNV cells transfected with HCVbp-coding plasmid.

**Figure S3**.

A. Detection of HCV core, E1, E2 of HCVbp after fractionation on a 20-60% sucrose gradient.

B. Effect of brefeldin A on HCV release.

C. HCV release required maturation of viral glycoproteins.

D. WNV, but not HCV, structural proteins *trans*-encapsidate WNV SG-replicon that is infectious in Huh-7.5 cells.

E. Effect of antiviral treatment of WNV SG-replicon on HCV release by BHK-WNV cells.

**Figure S4.**

A. Co-localization of 4cys-HCVbp with lipid droplets in BHK-WNV cells.

B. Pre-incubation of HCVbp with serum from HCV-cured patient abolished its infectivity.

C. Specificity of in-house HCV NS5A rabbit polyclonal antibody.

D. Infection of Huh-7.5 cells with HCVbp-4cys.

**Figure S5**.

A. Surface expression of human CD81 by HepG2-CD81 cells.

B. BHK-WNV cells, but not parental BHK-21, produced infectious HCVrp.

C. HCV receptors knockdown by siRNA in Huh-7.5 cells.

D. Inhibition of HCVrp entry by anti-CD81 and anti-SR-BI antibodies.

E. Inhibition of HCVrp entry by anti-HVR-1 antibodies.

F. Effect of NS2 on the buoyant densities and infectivity of HCVrp.

**Figure S6**. Control liver slices with secondary antibodies.

**Supporting Data**

**Supporting Table**

**Table S1**. Correlation between HCVrp infectivity and candidate receptor expression in several cell lines

**Supporting Materials and Methods**

**Supporting References**

**Supporting Data**

**HCV subgenomic replicon fails to promote the release of HCV particles**

We initially sought to express a plasmid encoding HCV structural genes of subtype 1a (from a cytomegalovirus immediate early promoter) in Huh-7.5 cells, a human hepatocellular carcinoma cell line selected for supporting high level replication of HCV RNA [10] with cell-adaptive mutations in both NS3 and NS5A [9]. A fraction of the HCV structural proteins was released in the supernatants (Fig. S1). However, the basal level of release was weak (a much larger proportion of supernatant than cell lysate was loaded on the gel) and inhibited by the presence in *trans* of a HCV subgenomic replicon of the same strain. This could possibly relate to the presence of cell culture adaptive mutations interfering with the assembly process, as previously shown for subtype 1b [19]. In addition, although Huh-7.5 cells support the production of JFH-1, packaging a reporter subgenomic replicon into infectious HCV particles required the presence in *trans* of a full-length HCV genome [76], i.e. not just a RNA encoding the structural genes. The existence of complex interactions between viral replication and production in these cells prompted us to look for alternative models, thus the use of BHK-WNV1 cells to test our hypothesis.

Various culture systems have been developed to study the biology of *Flaviviridæ*. Albeit budding into its lumen [77], HCV envelope proteins expressed in BHK-21 cells are retained in the ER [78], as similarly observed in Huh-7.5 cells [79]. Lack of release could relate to the absence of required viral factors such as HCV proteins, genomic components and/or replication-induced cellular changes. However, our attempts at stably establishing a HCV subgenomic replicon in these cells were unsuccessful, perhaps because factors required for persistent HCV replication were missing and/or others interfering.

**HCV particles released by BHK-WNV cells are not exosomes**

The release of particles containing HCV E1 and E2 proteins by non-human, non-hepatic cells has been reported upon human CD81 expression, and was interpreted as exosomes [80]. Heat shock protein-70 (Hsp70) is associated with exosomes [81], and our detection of Hsp70 in the supernatant of BHK-21 cells (plus or minus WNV subgenomic replicon) led us to consider such possibility as an alternative interpretation of the above-described results. However, the buoyant densities of the peaks of Hsp70 and HCV envelope proteins were different (Fig. S3A, lower panels), indicating that HCV particles originated from a different cellular compartment. In addition, the release of exosomes has been shown to be independent of brefeldin A-sensitive cellular traffic [81] and, indeed, we observed that this compound failed to affect the release of Hsp70-containing materials by BHK-WNV cells, whereas it blocked that of HCV particles (Fig. S3B). The distinctive glycosylation pattern displayed by secreted HCV envelope proteins (Fig. S3C) and lack of detection of HCV NS5A protein in the supernatant (not shown) also argued against cell debris as the explanation. These results strongly suggest that released HCV particles are distinct from exosomes.

**Study of HCV cell entry with BHK-WNV cell-produced reporter particles**

Significant insight into the HCV entry process has been gained through the use of the HCVpp system [40]; however, unlike the particles described herein, HCVpp differ from HCV virions in that they contain neither HCV core nor HCV genome. HCV entry has also been studied with HCVcc, but the reporter readout in such systems requires post-entry events, such as replication of the viral RNA and viral spreading, thus restricting analysis to cell types that are capable of supporting these processes [82]. Therefore, we adapted the present HCV particle-based system for quantitative, high throughput analysis of HCV entry *stricto sensu* into target cells, wherein the signal is independent of post-entry processes. We designed a system involving HCV reporter particles (HCVrp), wherein NS3 up to the last third of HCV NS5B in the HCVbp construct were replaced with the ORF of bacteriophage SP6 RNA polymerase (Fig. 5B). The corresponding plasmid was co-transfected along with the p2B system (Fig. 1B) into BHK-WNV cells to generate HCVrp, which upon each entry event would introduce an RNA molecule into the cytoplasm, resulting in production of some SP6 RNA polymerase. The target cells were then co-transfected with the p2B system plus a plasmid encoding EGFP fused with *Firefly* luciferase, linked to the T7 and SP6 promoters and an EMCV IRES (Fig. 5B). The target cells were treated with actinomycin D to decrease the background reporter gene expression in the absence of incoming SP6 RNA polymerase-encoding RNA.

With this system, *Firefly* luciferase (FFL) activity that increased in a dose-dependent fashion was detected in the cytoplasm of Huh-7.5 cells 24 hr after their incubation with HCVrp produced by BHK-WNV cells. In contrast, the FFL activity remained at background levels with materials released by parental BHK-21 cells co-transfected with the same plasmids (Fig. S5B). The reduced activity seen at the highest input likely reflects the presence of materials interfering with HCVrp entry only at high concentration. No *Renilla* luciferase activity was detected (data not shown), indicating that WNV subgenomic replicon was not packaged in infectious HCV particles, in confirmation with the quantitative analysis of particle-associated RNA described above (Fig. 3A).

The entry signal peaked with particles with buoyant densities of 1.13-1.15 g/cm^3^ (Fig. S5F). Since NS5A has been implicated in particle formation by interacting with core protein-coated lipid droplets [54] and was not expressed by this construct, the slightly heavier density displayed by HCVrp (compared to that of HCVbp) could relate to their lesser association with lipid droplets (Fig. S4B). A construct encoding HCV proteins up to NS2 (Fig. S5F, left panel) elicited a slightly higher viral entry signal than one without NS2 (Fig. S5F, right panel), in line with its role in HCV assembly in hepatic cells [83-86]. The discrepancy in magnitude with HCVcc could relate to post-entry events (amplification by viral replication and spreading). Lack of requirement for HCV replication allowed us to test several cell lines to obtain a correlation between expression of candidate receptors and viral entry (Supplemental Table S1).

**Supporting Materials and Methods**

**Glycosylation of HCV envelope proteins and inhibitors of HCV secretion.** Parental BHK and BHK-WNV cells were transfected with pHCVp7 plasmid and three days later, culture media was harvested, clarified as above and pelleted through 15% sucrose cushion (100,000 x *g*, for 3 hr) at 4º C. Cell lysates and pelleted supernatant were then incubated with endo-H (New England Biolabs) according to the manufacturer’s protocols. For brefeldin A (BFA) treatment, cells were transfected with pHCVp7 and, 12 hr later, culture medium was replaced with D-MEM containing 20 μM BFA (Sigma)*.* Cells were grown for another 24 hr and supernatant was harvested and analyzed by Western blot.

**RNA analysis and RT-qPCR.** Total RNA from sucrose fractions was extracted with Trizol LS (Invitrogen). RT-qPCR (TaqMan) of HCV 5’-UTR RNA was performed with QuantiTect Probe PCR kit (Qiagen) using IVT RNA standard corresponds to the HCV 5’-UTR. Briefly, nt 1-587 of p90HCVconFLlongpU was cloned into pCRII (Invitrogen) downstream of T7 promoter, resulted in pCRII-5’UTR. Plasmid was linearized with *Kpn* I, ethanol precipitated and used as DNA template for IVT RNA standard using T7 MEGAScript (Ambion). After Turbo DNAse treatment and acid phenol extraction, IVT RNA standard was resuspended in RNA storage solution (Ambion). For cDNA synthesis, IVT RNA standards were diluted 10-folds ranging from 1 ng to 1 fg in H_2_O. RT was performed with Superscript III (Invitrogen) at 50°C, for 1 hr, with p322 reverse primer (CTC CCG GGG CAC TCG CAA GC). One-tenth of cDNA products were used for TaqMan PCR using p44 forward (CCT GTG AGG AAC TAC TGT CTT CA) and p265 reverse (AAC ACT ACT CGG CTA GCA GTC TT) primers, together with a dual-labeled probe (CAL Fluor Gold 540 fluorophore-TCT GCG GAA CCG GTG AGT ACA-BHQ-1 quencher) (Biosearch Technologies, CA). TaqMan PCR was carried out at 94°C (15 sec), 56°C (30 sec), and 76°C (30 sec) for 45 cycles on a 7900HT thermocycler (Applied Biosystems).

To analyze HCV RNA in Huh-7.5 cells after infection with HCVbp: Huh-7.5 cells (5,000 cells/well) were seeded in triplicate on collagen-coated 96-well plates (BD BioCoat). The next day, cells were infected with aliquots of sucrose fractions containing HCVbp for 3 hr; after virus inoculum removal, cells were allowed to recover for another 3 hr, then transfected with pcDNA 3.1-based plasmid encoding HCV core-NS2, or, pcDNA 3.1 empty vector. HCV RNA was analyzed directly from infected cells harvested daily until day 5 using lysis buffer of TaqMan® Gene Expression Cells-to-CT™ kit (Ambion, Applied Biosystems, Invitrogen). Briefly, cells were washed once with 150 μl ice-cold PBS, then dissociated with 25 μl TrypLE (Invitrogen) for 15 min at 37º C. After inactivation of TrypLE with D-MEM, cells were pelleted (1500 rpm, 10 min, 4º C), then washed with ice-cold PBS (1500 rpm, 10 min, 4º C) and stored at -80º C. RT step was performed directly from cell lysates according to the manufacturer’s protocol, followed by TaqMan PCR with some modifications. Briefly, PCR was performed with HCV specific primers and probe as above; the PCR condition was 50º C for 2 min, 95º C for 10 min, then 95º C, 15 sec; 55º C, 30 sec; 72º C, 30 sec for 45 cycles.

For HCV and WNV RNA analysis from BHK-WNV cells: Total RNA was extracted from cells and pelleted supernatants as above followed by RT using random hexamer and Superscript III at 50º C, for 1 hr. qPCR was performed with HCV specific primers as above, or, *Renilla* luciferase-specific primers as the target gene for WNV-SG rep RNA.

**Determination of TCID_50_ of HCVbp (or HCVcc).** The supernatants of HCVbp-expressing BHK-WNV cells (or HCVcc-infected Huh-7.5 cells) were filtered through 0.45 µm PVDF membranes, then concentrated using 10^6^ MWCO Vivaspin filters (Sartorius Stedim, Gottingen, Germany). The concentrated particles (viral stock) were serially diluted and 100 µl of each dilution was incubated in sextuplicate for 2-3 hours with Huh-7.5 cells seeded on 96-well collagen-coated plates at 3-4 x 10^3^ cells/well, and cultured for an additional 3-4 days. Cells were rinsed with PBS, fixed with ice cold methanol:acetone (50:50 v:v) for 2 minutes, sequentially washed with increasing dilutions of ethanol (100-0%) in PBS (0-100%), and incubated with PBS containing 3% BSA and 0.2% Triton-X100 for 2 hr. The number of positive wells was determined using the Odyssey In-cell Western system (Li-Cor Biosciences, Lincoln, NE). Briefly, cells were incubated overnight with serum from an HCV-cured patient (1/400) and monoclonal antibodies against HCV structural genes (1-5 µg/ml) or affinity-purified rabbit IgG against NS5A (1-4 µg/ml); the cells were then washed and incubated for 1 hr with IRDyes®800CW- and 700DX-coupled Donkey IgG (Rockland Immunochemicals, Inc, Gilbertsville, PA). After the final wash, signal was analyzed using the Odyssey infrared imaging system. A well was considered as infected when macroscopically (84-µm resolution) yielding consistent signals at both wavelengths (800 and 700 nm), whose intensities were at least twice that of uninfected controls (minus the background). Control human serum, mouse isotype or non-immune rabbit IgG did not yield a positive signal. The dilution yielding 50% infected wells (TCID_50_) was determined after plotting the number of infected wells at each dilution on a log-linear graph, and was calculated using the Poisson’s law: the probability of a well not to be infected after inoculation with n pfu is: P_0_ = e^-n^; hence, when 50% of the wells are not infected, they have in average been inoculated with: n = 0.7 pfu/well (P_0_ = e^‑0.7^ = 0.5). With 100-µl inoculums, the TCID_50_ in the original sample is therefore 7 pfu/ml divided by both the dilution of the stock yielding 50% infected wells and the original sample over viral stock volume ratio. The same procedure was used for HCVcc titration using human serum and affinity-purified IgG raised in rabbit against JFH-1 NS5A or NS5B (a gift from Drs T. Wakita and T. Kato, National Institute of Infectious Diseases, Tokyo, Japan).

**Transduction of HepG2 cells with hCD81-lentivirus.** Total RNA was extracted from Huh-7.5 cells, and CD81 cDNA was synthesized with gene specific primer using Superscript RT III (Invitrogen) at 55°C, followed by PCR to amplify CD81 gene. The PCR product was cloned into pENTR 2B (Invitrogen) and this vector was used for recombination with pLenti6.2/V5-DEST (Invitrogen). Recombinant lentivirus-hCD81 was produced using ViraPower Lentiviral Expression Systems (Invitrogen). Lentivirus-containing supernatant was harvested at 72 hr post-transfection and filtered through PVDF 0.45 μm membrane and viral stock was stored at -80°C. To transduce HepG2 cells with hCD81-lentivirus, 5 x 10^5^ cells were seeded in a 6-well tissue culture plate and the next day were incubated with 100 μl virus inoculum in medium containing 10 μg/ml Polybrene. Three days later, fresh medium containing 2 μg/ml Blasticidin was added and medium further replaced every 3-4 days. Antibiotic-resistance colonies were expanded and analyzed for CD81 expression by flow cytometry with JS-81 mAb.

Parental HepG2 and HepG2-hCD81 cells were seeded on collagen-coated 8-well chamber slides and, the next day, were incubated with HCVbp for 2 hour. After virus inoculum removal, cells were further grown for 48 hours, then fixed, permeabilized and analyzed for NS5A expression by confocal microscopy.

**Production of tetracysteine (4cys)-tagged-HCV.** A variant of HCVbp (designated HCV-4Cys) was obtained by inserting within NS5A a tetracysteine tag that binds permeant bi-arsenical dyes that then become fluorescent [72]. After three-day infection with HCVbp-4Cys, incubation of the cells with the dyes (without cell fixation) elicited a specific staining predominantly in the perinuclear area (Fig. S4D, center and right panels); this staining was not observed with control, uninfected cells (Fig. S4D, left panel). We interpret these results as a reflection of the accumulation of tag-bearing protein resulting from RNA replication and translation.

**HCVrp entry assay with the reporter system.** Previous validation of amplified reporter system was achieved either after transfecting target cells with T7pol RNA produced in Huh-7 cells infected with vTF7-3, a recombinant vaccinia virus encoding T7pol [94] or, more directly, with recombinant T7pol using ProteoJuice (Novagen) (not shown). The leakiness of the system, i.e. the detection of FFL activity in the absence of incoming RNA, was greatly reduced by incubating the target cells with very low concentrations of actinomycin D following their transfection, without blocking the readout of specific incoming signal. One day before the assay Huh-7.5 or other tested cells were seeded at a density of 2.5-4 x 10^4^ cells/well in 96-well opaque white plates (Nunclon). Unless otherwise specified, infectivity assays were performed in triplicates. HCVrp were pelleted, then serially diluted in Opti-MEM I, then added onto the cells and incubated at 37º C for an hour; for some experiments, HCVrp were fractionated on sucrose gradient before incubation. After virus inoculum removal, cells were transfected with the p2B system and the reporter plasmid using Lipofectamine LTX and Plus reagent for 4 hr. Cells were then incubated in complete medium containing 0.05µg/ml actinomycin D (added to reduce background of amplified reporter system, generally < 1% of maximum signal) and were further incubated at 37º C for 20-24 hours. Cells were then washed once with ice-cold PBS and lysed with 20 µl 1 x Glo Lysis Buffer (Promega). *Firefly* luciferase (FFL) activities were measured in each well using 50 µl Bright-Glo Luciferase Substrate (Promega) using a Clarity luminescence plate reader (Biotek).

**Production of WNV reporter particles.** To produce WNVrp, BHK-WNV cells were transfected with pIRES1hyg-WNV. Three days later, WNVrp were harvested by centrifugation through 15% sucrose cushion at 100,000 x *g* for two and half hours; the resuspended pellet was used for infection of Huh-7.5 cells. After two days, RNL activity in target cells was measured using the *Renilla* Luciferase Assay System (Promega) using a Clarity luminescence plate reader (Biotek), or using the live-cell/permeant RNL substrate Enduren (Promega) over a period of 1 to 3 days.

**Huh-7.5 cell gene knockdown using siRNAs.** Huh-7.5 cells (3 x 10^5^) were seeded in 6-well plates and the next day were transfected with 100 nM siRNA (Dharmacon) targeting either CD81, SR-BI, Claudin-1, or ASGPR subunits 1 and 2, following the manufacturer’s instruction. Cells were also transfected with non-target siRNA and siGlo (Dharmacon) to test the specificity and efficiency of siRNA delivery, respectively. The efficiency of siRNA to knock down the intended target gene was confirmed 3 days post-transfection by flow cytometry (CD81, ASGPR and siGlo) or Western Blot (Claudin-1 and SR-BI). siRNA-treated cells were dissociated at 48 hr post transfection and seeded in 96-well plate at 5 x 10^4^ cells/well. Twenty-four hours later, cells were incubated with HCVrp for 1 hr, then transfected with the reporter system. The effect of siRNA in inhibiting HCV entry was assessed by the reduction of FFL activity compared to the control cells the next day.

**Supporting References**

76. Steinmann E, Brohm C, Kallis S, Bartenschlager R, Pietschmann T (2008) Efficient trans-encapsidation of hepatitis C virus RNAs into infectious virus-like particles. J Virol 82: 7034-7046.

77. Blanchard E, Brand D, Trassard S, Goudeau A, Roingeard P (2002) Hepatitis C virus-like particle morphogenesis. J Virol 76: 4073-4079.

78. Dubuisson J, Hsu HH, Cheung RC, Greenberg HB, Russell DG, et al. (1994) Formation and intracellular localization of hepatitis C virus envelope glycoprotein complexes expressed by recombinant vaccinia and Sindbis viruses. J Virol 68: 6147-6160.

79. Rouillé Y, Helle F, Delgrange D, Roingeard P, Voisset C, et al. (2006) Subcellular localization of hepatitis C virus structural proteins in a cell culture system that efficiently replicates the virus. J Virol 80: 32-41.

80. Masciopinto F, Giovani C, Campagnoli S, Galli-Stampino L, Colombatto P, et al. (2004) Association of hepatitis C virus envelope proteins with exosomes. Eur J Immunol 34: 2834-2842.

81. Lancaster GI, Febbraio MA (2005) Exosome-dependent trafficking of HSP70: a novel secretory pathway for cellular stress proteins. J Biol Chem 280: 23349-23355.

82. Koutsoudakis G, Kaul A, Steinmann E, Kallis S, Lohmann V, Pietschmann T, Bartenschlager R (2006) Characterization of the early steps of hepatitis C virus infection by using luciférase reporter viruses. J Virol 80: 5308-5320.

83. Jones CT, Murray CL, Eastman DK, Tassello J, Rice CM (2007) Hepatitis C virus p7 and NS2 proteins are essential for production of infectious virus. J Virol 81: 8374-8383.

84. Jirasko V, Montserret R, Lee JY, Gouttenoire J, Moradpour D, et al. (2010) Structural and functional studies of nonstructural protein 2 of the hepatitis C virus reveal its key role as organizer of virion assembly. PLoS Pathog 6: e1001233.

85. Ma Y, Anantpadma M, Timpe JM, Shanmugam S, Singh SM, et al (2011) Hepatitis C virus NS2 protein serves as a scaffold for virus assembly by interacting with both structural and nonstructural proteins. J Virol 85: 86-97.

86. Stapleford KA, Lindenbach BD (2010) Hepatitis C virus NS2 coordinates virus particle assembly through physical interactions with the E1-E2 glycoprotein and NS3-NS4A enzyme complexes. J Virol (In press).

87. Ploss A, Evans MJ, Gaysinskaya VA, Panis M, You H, et al (2009) Human occludin is a hepatitis C virus entry factor required for infection of mouse cells. Nature 457: 882-886.

88. Wadsack C, Hirschmugl B, Hammer A, Levak-Frank S, Kozarsky KF, et al (2003) Scavenger receptor class B, type I on non-malignant and malignant human epithelial cells mediates cholesteryl ester-uptake from high density lipoproteins. Int J Biochem Cell Biol 35: 441-454.

89. Huang T, Deng H, Wolkoff AW, Stockert RJ (2002) Phosphorylation-dependent interaction of the asialoglycoprotein receptor with molecular chaperones. J Biol Chem 277: 37798-37803.

90. Stewart A, Ham C, Zachary I (2002) The focal adhesion kinase amino-terminal domain localises to nuclei and intercellular junctions in HEK 293 and MDCK cells independently of tyrosine 397 and the carboxy-terminal domain. Biochem Biophys Res Comm 299: 62-73.

91. Bordin M, D'Atri F, Guillemot L, Citi S (2004) Histone deacetylase inhibitors up-regulate the expression of tight junction proteins. Mol Cancer Res 2: 692-701.

92. Me CJ, Harris HJ, Farquhar MJ, Wilson G, Reynolds G, et al (2009) Polarization restricts hepatitis C virus entry into HepG2 hepatoma cells. J Virol 83: 6211-6221.

93. Schmitt M, Horbach A, Kubitz R, Frilling A, Häussinger D (2004) Disruption of hepatocellular tight junctions by vascular endothelial growth factor (VEGF): a novel mechanism for tumor invasion. J Hepatol 41: 274-83.

94. Fuerst TR, Niles EG, Studier FW, Moss B (1986) Eukaryotic transient-expression system based on recombinant vaccinia virus that synthesizes bacteriophage T7 RNA polymerase. Proc Natl Acad Sci USA 83: 8122-8126.
